# Cytotoxic flavone-*C*-glycosides from the leaves of *Dypsis pembana* (H.E.Moore) Beentje & J.Dransf., Arecaceae: in vitro and molecular docking studies

**DOI:** 10.1186/s12906-023-04046-0

**Published:** 2023-06-30

**Authors:** Mohamed S. Abdelrahim, Afaf M. Abdel-Baky, Soad A. L. Bayoumi, Shaymaa M. Mohamed, Wael M. Abdel-Mageed, Enaam Y. Backheet

**Affiliations:** 1 Department of Pharmacognosy, Faculty of Pharmacy, Assiut University, Assiut, 71526 Egypt; 2 Department of Pharmacognosy, College of Pharmacy, King Saud University, Riyadh, 11451 Saudi Arabia

**Keywords:** *Dypsis pembana*, *Chrysalidocarpus pembanus*, Arecaceae, Flavonoids, Flavonoid-*C*-glycoside, Vicenin-II, Kaempferol-3-*O*-neohesperidoside, HepG-2

## Abstract

**Background:**

Cancer poses a health threat, with an increased incidence worldwide. Thus, it is essential to develop new natural anticancer agents. *Dypsis pembana* (H.E.Moore) Beentje & J.Dransf (DP) is an ornamental plant belonging to the family Arecaceae. This study aimed to isolate and identify phytoconstituents from the leaves of this plant and evaluate their in vitro cytotoxic activities.

**Methods:**

Different chromatographic techniques were applied to fractionate the hydro-alcoholic extract of DP and separate the major phytoconstituents. The isolated compounds were structurally elucidated based on their physical and spectroscopic data. The in vitro cytotoxic activities of the crude extract and fractions thereof were evaluated against human colon carcinoma (HCT-116), human breast carcinoma (MCF-7), and human hepatocellular carcinoma (HepG-2) cell lines via MTT assay. Moreover, selected isolates were tested against HepG-2 cell line. Molecular docking analysis was performed to investigate the interactions of these compounds with two potential targets, the human topoisomerase IIα and cyclin-dependent kinase 2 enzymes.

**Results:**

Thirteen diverse compounds were reported for the first time from DP, providing significant chemotaxonomic biomarkers. Among tested compounds, vicenin-II (**7**) was the most cytotoxic against HepG-2 cell line, with an IC_50_ value of 14.38 µg/mL, followed by isovitexin (**13**) (IC_50_ of 15.39 µg/mL). These experimental findings were complemented by molecular docking, which demonstrated that vicenin-II exhibited superior enzyme-binding affinities to the studied vital targets and shed light on the structure–activity relationships among the investigated flavone-*C*-glycosides members.

**Conclusion:**

The phytochemical profile of DP was characterized for the first time, reflecting chemotaxonomic data about the concerned species, genus, or even the family. Biological and computational findings revealed that vicenin-II and isovitexin are possible lead structures as inhibitors of the human topoisomerase IIα and cyclin-dependent kinase 2 enzymes.

**Supplementary Information:**

The online version contains supplementary material available at 10.1186/s12906-023-04046-0.

## Background

Globally, cancer is one of the leading causes of death. There are 200 varieties of cancer that have been described, each with distinct characteristics and treatments [1]. In Egypt, the most prevalent type of cancer is liver cancer, followed by breast cancer, while colorectal cancer is the seventh most common one [2]. *Dypsis* genus (family Arecaceae) comprises about 140 species. It is a palm with a wide range of habits, from big canopy trees to small shrubs, that is found in Madagascar and nearby islands [3]. Previous studies on the genus revealed the presence of diverse secondary metabolites; flavonoids, lignans, tannins, saponins, triterpenes, steroids and verified that the genus possess cytotoxic, antioxidant, antimicrobial, and hepatoprotective activities in addition to its ornamental importance [4, 5]. *Dypsis pembana* (H.E.Moore) Beentje & J.Dransf. is native to Pemba Islands, Ngezi Forest Reserve, Tanzania. Its synonym is *Chrysalidocarpus pembanus* H.E. Moore and popularly known as the Mpapindi Palm. It is widely used as an ornamental plant [6]. To date, no literature could be found about the phytochemical and biological investigation of this species. Therefore, the present investigation aimed to establish the phytochemical profile, explore chemotaxonomic characteristics, and evaluate the cytotoxic activity of this species. Additionally, molecular docking was used to assess the binding affinity of the isolated compounds towards certain proposed target enzymes.

## Materials and methods

### Plant material

As stated in our previous botanical study [7], DP leaves were collected from Al-Abed Palm Garden along Cairo-Alexandria Desert Road in March 2020. Dr. Trease Labib, a plant taxonomy consultant at the Egyptian Ministry of Agriculture certified the plant identity. A sample (Aun-Phg-0002016) has been provided to the herbarium of Pharmacognosy Department, Faculty of Pharmacy, Assiut University.

### General experimental procedure

The NMR spectra were recorded on Bruker Avance DRX spectrometer at 400 MHz and 500 MHz (Bruker Scientific Instruments, MA, USA) using CDCl_3_ and DMSO-*d*_*6*_ (Sigma, St. Louis, Mo., USA). For Column chromatography (CC), several adsorbents were used, including Polyamide SC-6 (Macherey-Nagel, Düren, Germany), silica gel G_60_ (60–120 mesh, Merck, Darmstadt, Germany), reversed phase RP-18 silica gel (E-Merck, Darmstadt, Germany), Sephadex LH-20 (25–100 mm mesh size, E-Merck, Darmstadt, Germany) and Diaion^®^ HP-20 (Sorbent Technologies, Norcross, GA, USA). Pre-coated silica G_60_ F_254_ 0.25 mm and RP-18 F_254_ 0.25 mm (E-Merck, Darmstadt, Germany) were used for TLC. *n*-hexane, dichloromethane (CH_2_Cl_2_), ethyl acetate (EtOAc), and methanol (MeOH) were obtained from El-Nasr Pharmaceutical and Chemical Co., Egypt. MCF-7, HepG-2, and HCT-116 cells were obtained from the American Type Culture Collection (ATCC, Rockville, MD). Dimethyl sulfoxide, vinblastine sulfate, MTT (3-(4,5-Dimethylthiazol-2-yl)-2,5-diphenyltetrazoliumbromide) and trypan blue dye were obtained from Sigma-Aldrich Chemical Co. (St. Louis, Mo., USA). Fetal bovine serum (Lonza, Belgium). RPMI-1640 (Roswell Park Memorial Institute) medium, DMEM and HEPES buffer solution, ʟ-Glutamine, Gentamycin, Trypsin-EDTA 0.25% were obtained from Lonza Bioscience (Belgium).

### Extraction and isolation

**Scheme 1:**
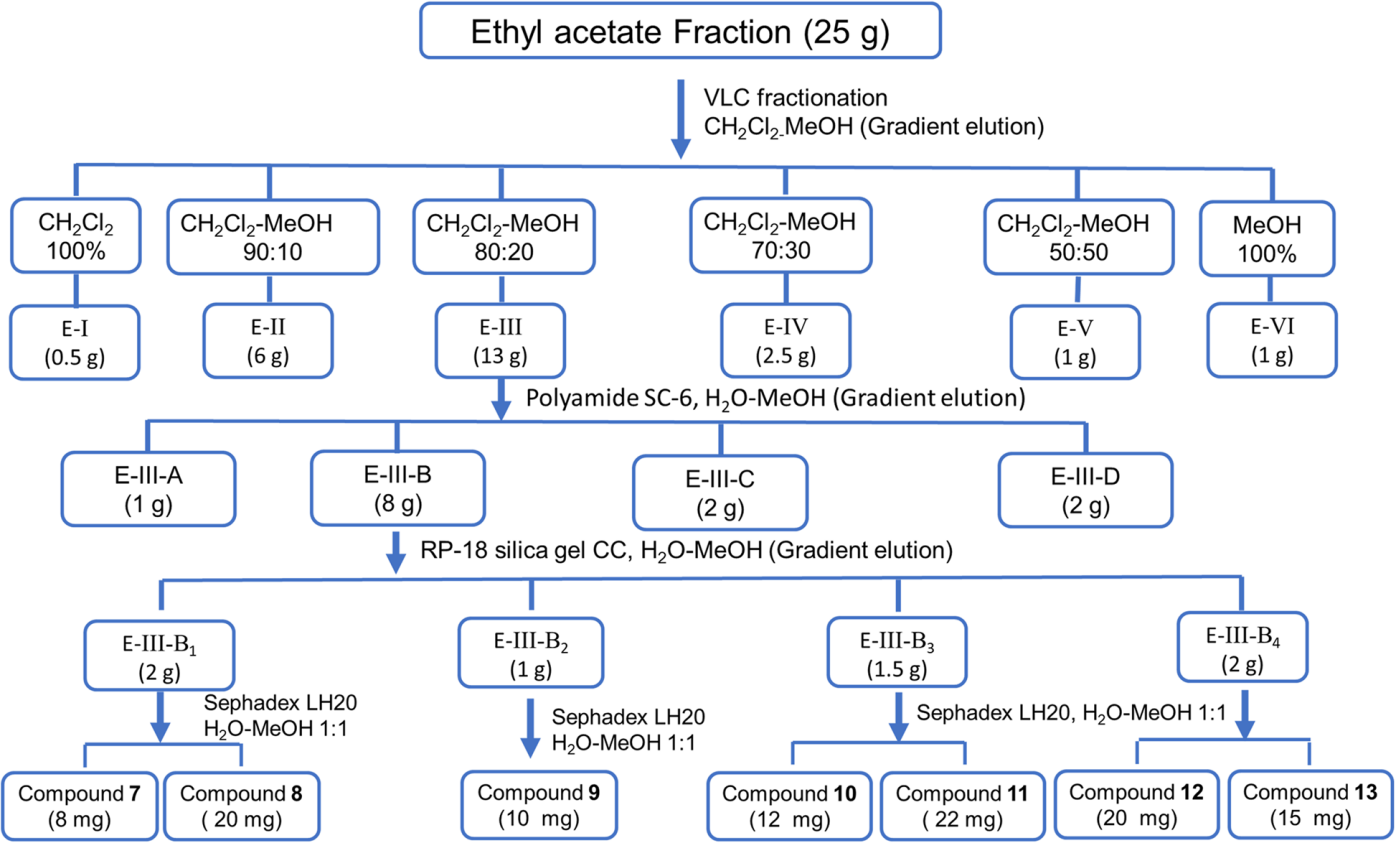
Isolation and purification of compounds **7**–**13** from the ethyl acetate fraction of *Dypsis pembana* leaves

Extraction of the leaves (5 kg) via successive maceration of the dried powder in 70% methanol (5 × 20 L) and vacuum concentration of extracts resulted in a crude residue of 870 g. For fractionation, a suspension of this crude extract in distilled H_2_O (500 mL) was sequentially partitioned with *n*-hexane (5 × 1 L), dichloromethane (CH_2_Cl_2_) (5 × 1 L), and ethyl acetate (EtOAc) (5 × 1 L) (supporting data, Scheme S1). Each phase was concentrated under reduced pressure to give the corresponding fractions: *n*-hexane fraction (64 g), CH_2_Cl_2_ fraction (37 g), EtOAc fraction (30 g), and aqueous fraction (700 g). As illustrated in (supporting data, Scheme S2), vacuum Liquid Chromatography (VLC) of a portion of the *n*-hexane fraction (25 g), using gradient of *n*-hexane-EtOAc mixtures for elution, gave six subfractions (H-I to H-VI). H-II (7 g) was subjected to silica gel CC (280 g), eluted with *n*-hexane-EtOAc 95:5, to afford **1** (15 mg) and **2** (20 mg). H-III (4.5 g) was fractionated using silica gel CC (180 g), which eluted with *n*-hexane-EtOAc 9:1, to yield **3** (100 mg), after purification with crystallization. A portion of the CH_2_Cl_2_ fraction (25 g) was subjected to silica VLC, eluted with gradient mixtures of CH_2_Cl_2_-MeOH. As a result, six subfractions (D-I to D-VI) were collected. D-IV (6 g) was chromatographed over silica CC (280 g), eluted with CH_2_Cl_2_-MeOH 9:1, then, re-chromatographed on silica gel CC (240 g), eluted gradient with CH_2_Cl_2_-MeOH solvent system to give three subfractions D-IV-1 to D-IV-3. D-IV-2 (200 mg) was subjected to Sephadex LH-20 CC (100 g), eluted with MeOH-H_2_O 1:1, to afford **4** (12 mg) and **5** (10 mg). Repeated silica gel chromatography of D-IV-3 (1.8 g) and further purification afforded **6** (100 mg). Schematic representation of the fractionation process of dichloromethane fraction was included (supporting data, Scheme S3). As depicted in the schematic diagram (Scheme 1), a portion of the EtOAc fraction (25 g) was fractionated via VLC, applying gradient mixtures of CH_2_Cl_2_-MeOH for elution, into six subfractions (E-I to E-VI). E-III (13 g) was loaded on polyamide SC-6 CC (250 g) and eluted with H_2_O-MeOH gradient mixtures to be fractionated into four subfractions E-III-A to E-III-D. Fractionation of E-III-B (8 g) with reversed phase silica gel CC (250 g), eluted with H_2_O-MeOH gradient mixtures, afforded four subfractions E-III-B_1_ to E-III-B_4_. E-III-B_1_ (2 g), E-III-B_2_ (1 g), E-III-B_3_ (1.5 g), and E-III-B_4_ (2 g) were separately subjected to Sephadex LH-20 CC (100 g), eluted with a combination of MeOH-H_2_O 1:1. As a consequence, E-III-B_1_ afforded **7** (8 mg) and **8** (20 mg), E-III-B_2_ afforded **9** (10 mg), E-III-B_3_ gave **10** (12 mg) and **11** (22 mg), and E-III-B_4_ resulted in **12** (20 mg) and **13** (15 mg).

### Cytotoxic assay

The in vitro cytotoxic activity of the crude extract and the derived fractions was evaluated by viability assay against HCT-116, MCF-7, and HepG-2 cell lines, and performed as described previously [8, 9]. Briefly, the cell lines were subcultured in a humidified 5% CO_2_ incubator twice to three times per week at 37 °C. The tumour cell lines were incubated at a density of 5 x 10^4^ cell/well. Then, from each investigated sample, three replicates of 8–10 concentrations were prepared and added; untreated controls with 0.5% DMSO were also included. The MTT assay was performed to assess the number of viable cells after 24 h of incubation. In brief, the medium in each well was withdrawn and replaced with a combination of fresh RPMI 1640 medium (100 μL) and 12 mM MTT stock solution (10 μL) and incubated for 4 h at 37 °C. After replacing a portion of the medium with 50 μl of DMSO, the optical density was estimated at 590 nm using the microplate reader to evaluate the cells viability after sample treatment. Using Graphpad Prism 8.0.1 software, IC_50_ values were calculated from graphic plots of the dose response curves.

### Statistical analysis

All experimental data are represented as mean ± standard deviation (SD) of three replicates. Microsoft Excel program 2016 was used for graphs drawing.

### Molecular docking

The target ligands for modelling were built using the builder interface of the MOE (Molecular Operating Environment) software package 2020.01 and subjected to conformational search. Conformers were optimized through energy minimization until a RMSD gradient of 0.01 Kcal/mol and RMS distance of 0.1 Å with MMFF94X force-field and the partial charges were automatically calculated. The obtained database was then saved as MDB file to be used in the docking investigation. The x-ray co-crystal structure of human topoisomerase IIα in complex with DNA and etoposide (PDB code: 5gwk) [10], and co-crystal structure cyclin-dependent kinase 2 in complex with sunitinib (PDB code: 3ti1) [11], were obtained from Protein Data Bank. Docking was run on the binding site of the co-crystallized ligand. Since the crystal structure contains a ligand molecule, the program automatically identifies the binding site, and we dock the tested ligands on it. For structure preparation, three steps were done: a. Correct: in which the program check atoms connections and corrects any break in the chains. b. Protonate 3D: in which the program adds the hydrogen atoms. c. Fixing the potential of the enzyme atoms after selection of the whole enzyme structure. Docking of the conformations database of the target ligands was done using MOE-DOCK software wizard. The following parameters were adjusted: Receptor and solvent as receptor, Co-crystalized ligand atoms as active site, Database containing test ligands as ligand, London dG as initial scoring function, GBVI/WSA dG as final scoring function, MMFF94x force field was used for calculating the energy parameters of the ligand – cleavage complex model. To compare between the conformers, London dG was used as scoring function, lower values indicate more favourable poses. The dock calculations were run, and the obtained poses were studied. The 2D and 3D ligand interactions for each compound were saved as picture files.

## Results

**Fig. 1:**
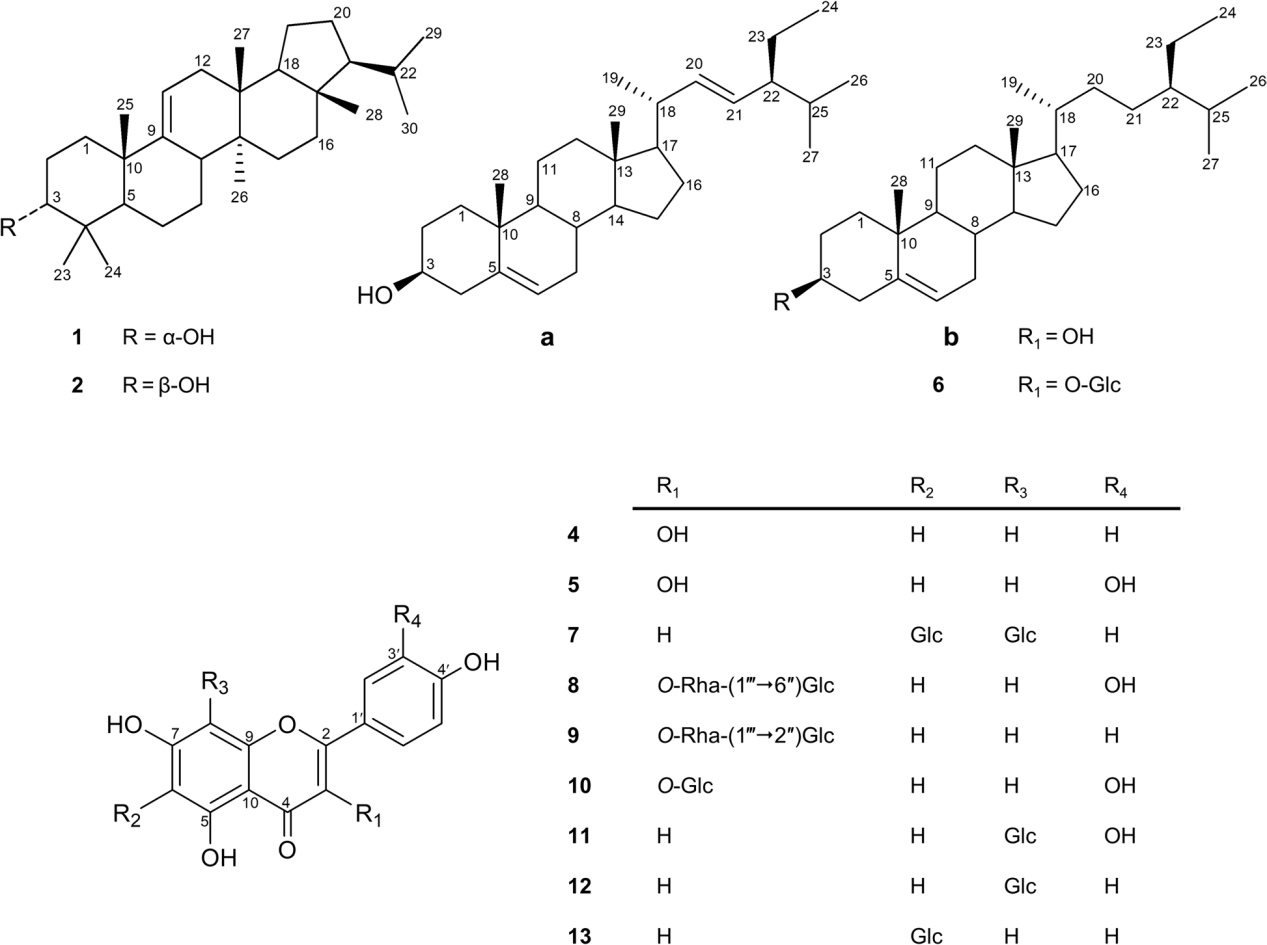
Structures of the compounds isolated from DP leaves

Using various chromatographic techniques and spectral analysis, thirteen compounds (Fig. 1) were isolated and identified from DP. Physicochemical properties, spectral data (^1^H and ^13^C NMR) of the isolated compounds (**1**–**13**) (supporting data, S1), and their spectra (supporting data, Fig. S1–S26) were included. These compounds were identified as arborinol (**1**) [12], isoarborinol (**2**) [13], stigmasterol (**3a**) [14], β-sitosterol (**3b**) [14]. Kaempferol (**4**) [15], quercetin (**5**) [15], β-sitosterol-3-*O*-β-d-glucopyranoside (**6**) [16], vicenin-II (**7**) [17], rutin (**8**) [18], kaempferol-3-*O*-neohesperidoside (**9**) [19], isoquercetrin (**10**) [20], orientin (**11**) [21], vitexin (**12**) [5], and isovitexin (**13**) [5].

Concerning the in vitro cytotoxic study, the percentages of cell viability were used to make the dose response curves of cytotoxic activity of the crude extract and different fractions (supporting data, Fig. S27), and estimate their IC_50_ values (Table 1) which revealed that the most potent fractions were the ethyl acetate and *n*-hexane fractions against HepG-2 cell line. Based on that, the cytotoxic activity of certain compounds isolated from these fractions was investigated against HepG-2 cell line, including two triterpenes (arborinol and isoarborinol) and four flavonoid-*C*-glycosides (vicenin-II, orientin, vitexin, and isovitexin), in a concentration-dependent manner. The percentage cell viability at ten different concentrations were used to prepare the dose response curves of these compounds (supporting data, Fig. S28) and to calculate IC_50_ values of the investigated samples which are listed in Table 1. The ethyl acetate fraction showed the highest activity against both HepG-2 and HCT-116 cell lines, with IC_50_ values of 42.2 and 59.9 µg/mL, respectively. Regarding MCF-7 cell line, the *n*-hexane fraction showed the highest cytotoxic activity (IC_50_ value of 113 µg/mL). Among investigated compounds, vicenin-II (**7**) and isovitexin (**13**) showed the highest cytotoxic activity, with IC_50_ values of 14.38 and 15.39 µg/mL, respectively.

**Table 1:** Cytotoxic activity (IC_50_ values) of the crude extract, fractions, and certain compounds of DP via MTT assay against HCT-116, MCF-7, and HepG-2

**Sample**	**HCT116**	**MCF-7**	**HepG-2**
	**[µg/mL]**		
**Crude extract**	248 ± 4.6	422 ± 9.1	316 ± 8.7
***n*-Hexane fraction**	85.4 ± 2.6	113 ± 2.7	60.1 ± 2.8
**DCM fraction**	111 ± 3.1	230 ± 4.2	170 ± 4.9
**EtOAc fraction**	59.9 ± 1.2	166 ± 3.9	42.2 ± 2.1
**Aqueous fraction**	> 500	> 500	> 500
**1**	-	-	106.39 ± 3.12
**2**	-	-	356.92 ± 10.27
**7**	-	-	14.38 ± 0.69
**11**	-	-	82.98 ± 4.06
**12**	-	-	47.93 ± 2.64
**13**	-	-	15.39±0.95
**Vinblastine sulfate**	2.30 ± 0.35	3.59 ± 0.43	3.01 ± 0.24

Results are expressed as mean ± SD which were derived from the dose response curve of triplicate analyses

**Fig. 2:**
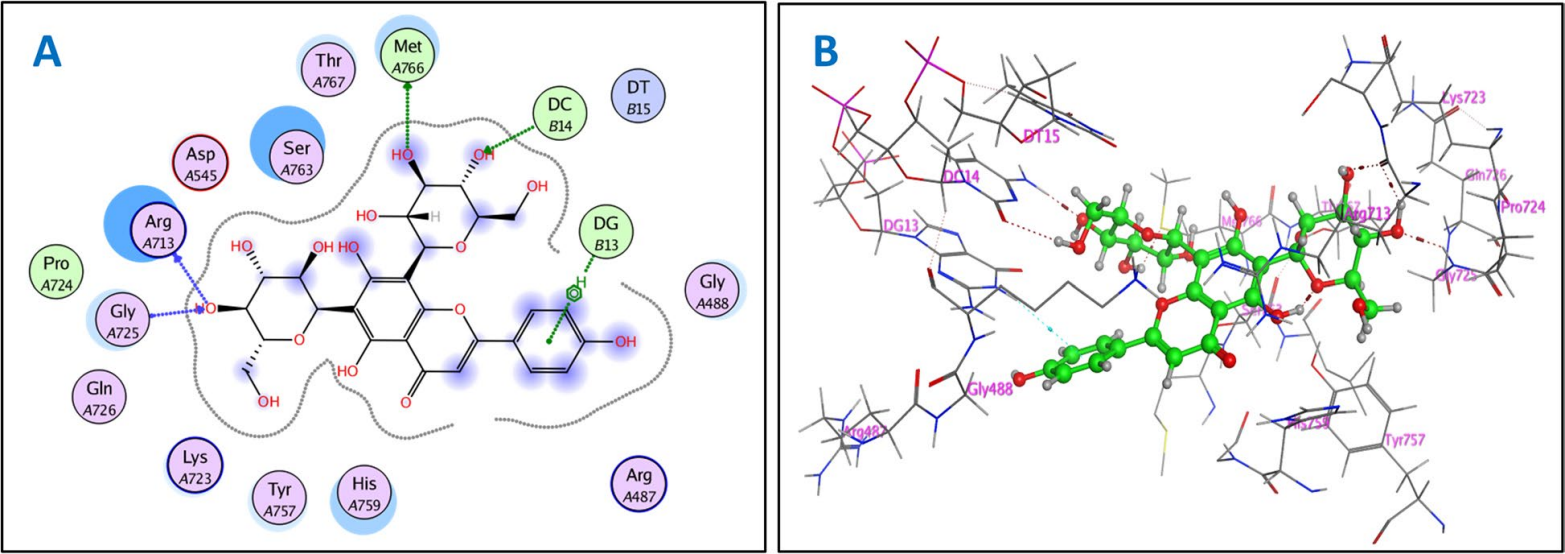
2D (**A**) and 3D (**B**) interactions of compound **7** with topoisomerase IIα (PDB ID: 5gwk)

The molecular docking study provided extensive insight into binding patterns for the investigated compounds in the active site of target enzymes and linked the observed in vitro activity with binding scores. Concerning topoisomerase IIα, docking protocol was validated by re-docking of the co-crystalized etoposide at the active site of topoisomerase IIα (PDB ID: 5gwk). The re-docking RMSD = 0.8637 Å and binding score = −7.67 kcal/mol. All the key interactions accomplished by the co-crystalized ligand with the key amino acids in the binding site were reproducible using the followed docking setup. The validated docking setup was then used to investigate the ligand-receptor interactions and binding patterns for the designed compounds which then compared to that of etoposide in its active site (Fig. 2, supporting data, Fig. S29–S31). The amino acid residues and nitrogenous bases of DNA involved in interaction at binding site with co-crystallized etoposide are Lys A723, Arg A713, Gly A462, Met A766, and DG B13 [22], where the main interaction is H-bonding with amino acid residues and nitrogenous bases. From docking investigation, all isolated compounds interact with the same amino acids as co-crystallized etoposide at different poses, where the common interactions among investigated compounds are H-bond with Met A766 and Arg A713 residues, respectively, with average length of 2.63 and 2.71 Å, H-bond or π-cation interactions with Lys A723 and H-bond with DNA bases DT B15 and DG B13. Beside previously mentioned main interactions, isolated compounds interacted with additional amino acids and nitrogenous bases of DNA as extra binding interactions which formed and mediated by H-bonding and π-H bonding interactions as shown in (supporting data, Table S1). These extra interactions played an important role in stabilizing formed ligand-enzyme-DNA complex and that justified lower binding scores of isolated compounds than etoposide, specifically compounds rutin (**8**) (H-bond with Ser A714 and Ile A856), kaempferol-3-*O*-neohesperidoside (**9**) (π-H bond with His A759) and vicenin-II (**7**) (H-bond with His A759 and Glu A461). The 2D and 3D interactions of the most active compound **7** with topoisomerase IIα (PDB ID: 5gwk) is shown in Fig. 2.

**Fig. 3:**
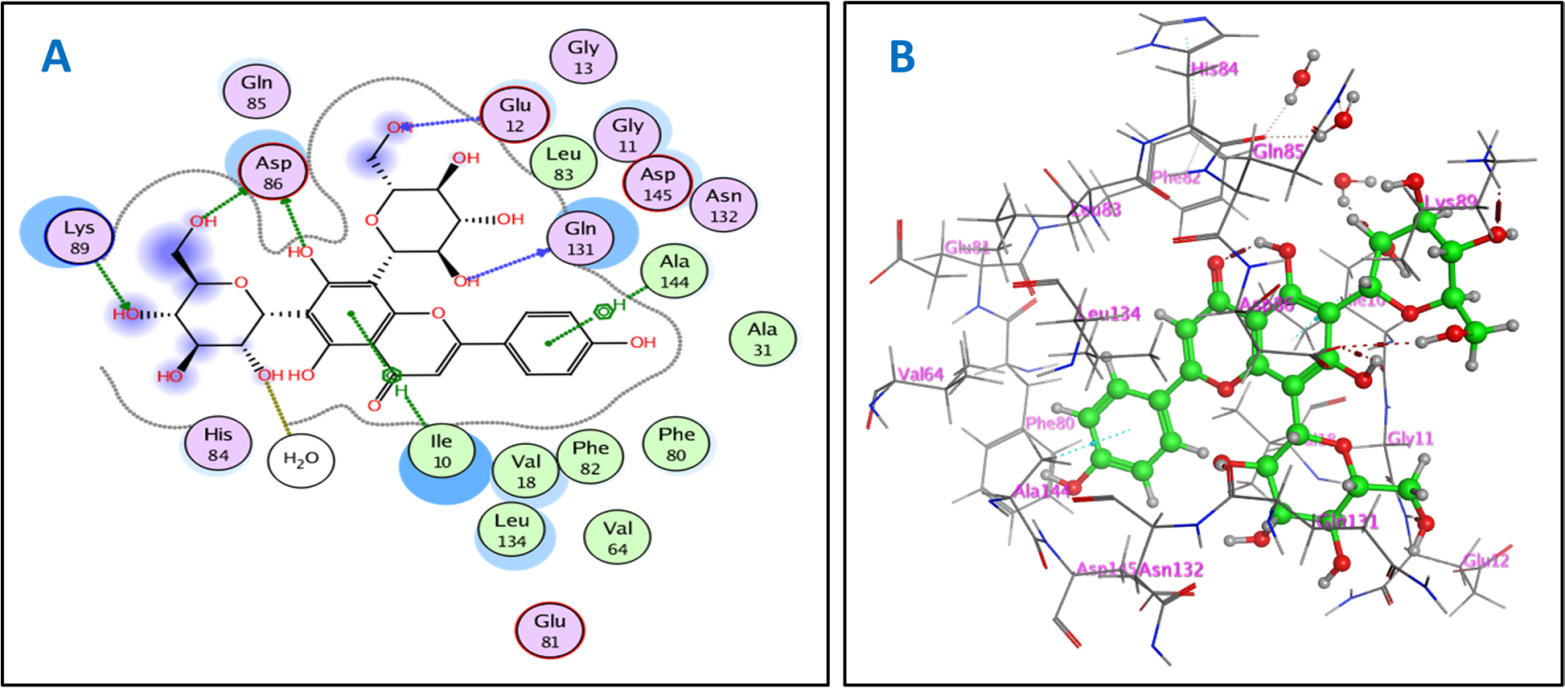
2D (**A**) and 3D (**B**) interactions of compound **7** with cyclin-dependent kinase 2 (PDB ID: 3ti1)

For cyclin-dependent kinase 2, docking protocol was validated by re-docking of the co-crystalized sunitinib at the active site of cyclin-dependent kinase 2 (PDB ID: 3ti1). The re-docking RMSD = 0.9424 Å and binding score = −7.62 kcal/mol. All the key interactions accomplished by the co-crystalized ligand with the key amino acids in the binding site were reproducible using the followed docking setup. The validated docking setup was then used to investigate the ligand-receptor interactions and binding patterns for the designed compounds which then compared to that of sunitinib in its active site (Fig. 3, supporting data, Fig. S32–S34). Studying the interaction between cyclin-dependent kinase 2 enzyme and co-crystallized sunitinib at active site revealed that amino acid residues involved in the binding are Asp86, Glu81, Ile10, and Ala144 [23] where the main interactions are H-bonding between with Asp86 with length equal to 1.67 Å, H-bond with Ile10 with length of 1.89 Å, H-bonding with Glu81 with length of 2.01Å and π-H bond with Ala144 residue. The docking investigation showed that most docked compounds interact with the most of amino acids involved in interaction with co-crystallized inhibitor sunitinib at different poses, but the common residues are Asp86, Ile10 and Ala144. Most investigated compounds formed H-bond with Asp86, Glu8, Gln131, and Lys89 residues, respectively, with average lengths of 1.81, 1.79, 1.75 and 2.13 Å and π-H bond with Ala144 and Ile10 residues. In addition to these main interactions, isolated compounds interacted with additional amino acids as extra binding interactions which mediated by H-bonding and π-H bonding interactions such as Glu8, Glu12, Leu83, and Asp145. These extra interactions contributed to lower binding scores of the isolated compounds than sunitinib which observed with compounds vitexin (**12**) (π-H bond with Val18), rutin (**8**) (H-bond with Glu12 and Leu298), isoquercetrin (**10**) (π-H bond with Val18 and H-bond with Glu81) and vicenin-II (**7**) (H-bond with Glu12 and Lys89), reflecting their higher binding affinity with cyclin-dependent kinase 2 enzyme, and the high probability of cytotoxic activity of the isolated compounds through inhibition of both cyclin-dependent kinase 2 and topoisomerase IIα enzymes. The 2D and 3D binding pattern of compound **7** with cyclin-dependent kinase 2 (PDB ID: 3ti1) is depicted in Fig. 3.

## Discussion

The phytochemical investigation resulted in thirteen compounds identified for the first time from DP, which could be employed for chemotaxonomic studies. The *n*-hexane fraction yielded three compounds (**1**–**3**). Compounds **1** and **2** were found to be arborane-type triterpenes, namely arborinol (**1**) and isoarborinol (**2**). Notably, they were isolated from the Arecaceae family member, *Acrocomia totai* [24]. Compound **3** was identified as a mixture of the two closely related sterols, stigmasterol (**3a**) and β-sitosterol (**3b**). Other three compounds were isolated from the CH_2_Cl_2_ fraction, and identified as kaempferol (**4**), quercetin (**5**), and β-sitosterol-3-*O*-β-d-glucopyranoside (6). The EtOAc fraction afforded seven compounds (**7**–**13**). Compounds **7**, **11**, **12**, and **13** were found to be a family of flavonoid-*C*-glycosides and identified as apigenin-6,8-di-*C*-β-d-glucopyranoside (vicenin-II), luteolin-8-*C*-β-d-glucopyranoside (orientin), apigenin-8-*C*-β-d-glucopyranoside (vitexin) and apigenin-6-*C*-β-d-glucopyranoside (isovitexin), respectively. These flavone-*C*-glycosides were previously isolated from two related species, *D. lutescens* [4] and *D. leptocheilos* [5]. Compounds **8**–**10** were identified as the flavonoid-*O*-glycosides, rutin, kaempferol-3-*O*-neohesperidoside, and isoquercetrin, respectively. These findings possess chemotaxonomic significance and offer various unique biomarkers since this is the first report for the isolation of eight compounds (**1**–**6**, **8** and **10**) from the genus *Dypsis* and the isolation of kaempferol-3-*O*-neohesperidoside (**9**) from family Arecaceae.

To our knowledge, this is the first time for investigation of vicenin-II against HepG-2. Supporting these findings, vicenin-II has inhibited the growth of prostate cancer (PC-3, DU-145 and LNCaP cells) in vitro [25, 26], inducing apoptosis in human colon cancer cell line (HT-29) [27] and inhibited cancer metastasis in lung adenocarcinoma cells [28]. Orientin showed cytotoxic activity against HepG-2 [29–31] and showed cytotoxic activity against HT-29 [32], human cervix carcinoma (HeLa) [33] and human bladder carcinoma cell lines (T24) [34]. Vitexin showed cytotoxic activity against HepG-2 (IC_50_ = 92.08 μg/mL) [35–39]. Moreover, it exhibited cytotoxic activity against rat brain tumor (C6), HeLa, HT-29 and African green monkey kidney epithelium (Vero) cell lines [40]. Isovitexin showed cytotoxic activity against HepG-2 (IC_50_ = 21.70 μg/mL) and showed cytotoxic activity against human laryngeal squamous cell carcinoma (Hep-2), HeLa and MCF-7 cell lines [41]. Arborinol and isoarborinol showed cytotoxic activity against HepG-2, with IC_50_ values of >100 μg/mL and IC_50_ = 96.41 μg/mL, respectively. Furthermore, these triterpenes showed cytotoxic activity against HeLa, human lung adenocarcinoma (LU-1), human breast cancer (MDA-MB-231) and human tubular adenocarcinoma (MKN7) cell lines [38]. The cytotoxic activity of the crude extract and four fractions were tested against HCT-116, MCF-7, and HepG-2 cell lines by MTT assay, using vinblastine sulfate as a standard in a concentration-dependent manner.

Our docking study with topoisomerase IIα showed that the in vitro cytotoxic activity of vicenin-II (**7**), orientin (**11**), vitexin (**12**), and isovitexin (**13**) against HepG-2 cell line went in line with the docking results. Since these flavonoid-*C*-glycosides vicenin-II (**7**), orientin (**11**), vitexin (**12**) and isovitexin (**13**) showed the lowest IC_50_ values of 14.38, 82.98, 47.93, and 15.39 µg/mL, respectively, which were consistent with low binding scores of these compounds on topoisomerase IIα enzyme that were −8.52, −7.83, −7.88, and −7.51 kcal/mol, respectively. Vicenin-II (**7**) showed the highest cytotoxic activity, reflected from its high binding affinity with topoisomerase IIα and promising IC_50_ value of 14.38 µg/ml. This activity could be justified by observations that 4''' OH group of sugar at C-6 forms H-bond with Gly A729 and Arg A713, 3'' OH and 4'' OH groups of sugar at C-8 form H-bonds with Met A766 and DC B14, respectively. Furthermore, aromatic ring B forms π-H bond with DG B13. While isovitexin (**13**) showed lower binding affinity than vicenin-II (**7**) and that explained by the lack of sugar moiety at C-8, but it still has higher promising activity than those of orientin (**11**) and vitexin (**12**), because the sugar moiety exists at C-6, while in orientin (**11**) and vitexin (**12**) present at C-8, and 3''' OH of C-6 sugar forms H-bond with Lys A489 and DT B15. In addition, aromatic ring B forms π-cation bond with Lys A723 and 4' OH group forms H-bond with Asn A770 (Aspragine) residue. Both orientin (**11**) and vitexin (**12**) exerted the same interactions, but vitexin (**12**) had lower binding score and IC_50_ values and that due to H-bond formation between 4' OH of aromatic ring B and DG B13. Furthermore, vicenin-II (**7**) showed the highest cytotoxic activity indicated from its lower binding score with cyclin-dependent kinase 2 and promising IC_50_ value of 14.38 µg/mL, which attributed to H-bonds between 4''' OH and 6''' OH groups of C-6 sugar with Lys89 and Asp86, respectively. Moreover, 6'' and 2'' OH groups form H-bond with Glu12 and Gln131, and aromatic ring B forms π-H bond with Ala144 residue. Where isovitexin (**13**) showed the same interactions as vicenin-II (**7**), with exception of H-bonds with Glu12 and Gln131 due to absence of sugar moiety at C-8 and that justified lower binding affinity and biological activity of isovitexin (**13**) than vicenin-II (**7**). Vitexin (**12**) had higher activity than orientin (**11**) because vitexin (**12**) showed interactions similar to that of vicenin-II (**7**) such as H-bonds with Lys89 and Gln131 and π-H bond with Ala144 residue. The docking of the isolated compounds gave good binding scores which were consistent with their in vitro cytotoxic activity against HepG-2 cell line. Specifically, compounds vicenin-II (**7**), orientin (**11**), vitexin (**12**) and isovitexin (**13**) that had IC_50_ values of 14.38, 82.98, 47.93 and 15.39 µg/mL, respectively, were highly correlated with their binding scores that were −8.67, −7.65, −8.31 and −8.12, kcal/mol, respectively.

## Conclusions

Thirteen compounds were first reported from *Dypsis pembana*. Vicenin-II and isovitexin, two flavonoid-*C*-glycosides, demonstrated promising cytotoxic activities against HepG-2 cell line, offering lead scaffold for further development of modified structures with enhanced activity. It was shown that one of the key elements affecting these flavonoid-*C*-glycosides' ability to exert cytotoxic effects is the number and location of their sugar moieties. Thus, additional in vivo studies are required to confirm the cytotoxic action of these compounds.

## Supplementary Information

**Additional file 1.**

## Data Availability

All data generated or analyzed during this study are included in this published article and its supplementary information file.

## Abbreviations

HCT-116: Human colon carcinoma cell line
MCF-7: Human breast carcinoma cell line
HepG-2: Human hepatocellular carcinoma cell line
MTT: 3-(4,5-Dimethylthiazol-2-yl)-2,5-diphenyltetrazoliumbromide
VLC: Vacuum Liquid Chromatography
CC: Column Chromatography
IC_50_: 50% inhibitory concentration
SD: Standard deviation
MOE: Molecular Operating Environment
HT-29: Human colon cancer cell line
HeLa: Human cervix carcinoma cell line
CH_2_Cl_2_: Dichloromethane
EtOAc: Ethyl acetate
