# Supplementary material for: Cytotoxic flavone-C-glycosides from the leaves of Dypsis pembana (H.E.Moore) Beentje & J.Dransf., Arecaceae: in vitro and molecular docking studies
Source: BMC Complement Med Ther. 2023 Jun 30;23:214. doi: 10.1186/s12906-023-04046-0 (PMC10311779; doi:10.1186/s12906-023-04046-0)
Supplement: Supplementary file 1 — Additional file 1. [file 12906_2023_4046_MOESM1_ESM.pdf]

Cytotoxic flavone-*C*-glycosides from the leaves of *Dypsis pembana* (H.E.Moore) Beentje & J.Dransf., Arecaceae: in vitro and molecular docking studies

Mohamed S. Abdelrahim[1*], Afaf M. Abdel-Baky[1], Soad A.L. Bayoumi[1], Shaymaa M. Mohamed[1], Wael M. Abdel-Mageed[1,2] and Enaam Y. Backheet[1]

Affiliation

[1]Department of Pharmacognosy, Faculty of Pharmacy, Assiut University, Assiut 71526, Egypt

[2]Department of Pharmacognosy, College of Pharmacy, King Saud University, Riyadh 11451, Saudi Arabia

[*]Corresponding author

Mohamed S. Abdelrahim, E-mail: mohamed.salah12@pharm.aun.edu.eg

Other authors emails :

Afaf M. Abdel-Baky: afaf.abdelaziz@pharm.aun.edu.eg

Soad A.L. Bayoumi: soad.bayoumi@pharm.aun.edu.eg

Shaymaa M. Mohamed: shaymaa.makram@aun.edu.eg

Wael M. Abdel-Mageed: wabdelmageed@ksu.edu.sa

Enaam Y. Backheet: enaam@pharm.aun.edu.eg

Contents

List of schemes

Scheme S1: Extraction and fractionation of *Dypsis pembana* leaves 4

Scheme S2: Isolation and purification of compounds **1**–**3** from the *n*-hexane fraction of *Dypsis pembana* leaves 4

Scheme S3: Isolation and purification of compounds **4**–**6** from the dichloromethane fraction of *Dypsis pembana* leaves...5

List of data

S 1 Physicochemical and spectral data of the isolated compounds (**1**–**13**)...............................4

List of figures

Fig. S1 ^{1}H NMR spectrum of compound **1** ($CDCl_3$, 400 MHz)..10

Fig. S2 DEPT-Q spectrum of compound **1** ($CDCl_3$, 100 MHz)..10

Fig. S3 ^{1}H NMR spectrum of compound **2** ($CDCl_3$, 400 MHz)...11

Fig. S4 DEPT-Q spectrum of compound **2** ($CDCl_3$, 100 MHz)..11

Fig. S5 ^{1}H NMR spectrum of compound **3** ($CDCl_3$, 400 MHz)...12

Fig. S6 ^{13}C NMR spectrum of compound **3** ($CDCl_3$, 100 MHz)..12

Fig. S7 ^{1}H NMR spectrum of compound **4** (DMSO-d_6, 400 MHz). 13

Fig. S8 ^{13}C NMR spectrum of compound **4** (DMSO-d_6, 100 MHz). 13

Fig. S9 ^{1}H NMR spectrum of compound **5** (DMSO-d_6, 400 MHz). 14

Fig. S10 ^{13}C NMR spectrum of compound **5** (DMSO-d_6, 100 MHz). 14

Fig. S11 ^{1}H NMR spectrum of compound **6** (DMSO-d_6, 400 MHz). 15

Fig. S12 ^{13}C NMR spectrum of compound **6** (DMSO-d_6, 100 MHz). 15

Fig. S13 ^{1}H NMR spectrum of compound **7** (DMSO-d_6, 400 MHz). 16

Fig. S14 ^{13}C NMR spectrum of compound **7** (DMSO-d_6, 100 MHz). 16

Fig. S15 ^{13}C NMR spectrum of compound **8** (DMSO-d_6, 125 MHz). 17

Fig. S16 ^{1}H NMR spectrum of compound **8** (DMSO-d_6, 500 MHz). 17

Fig. S17 ^{1}H NMR spectrum of compound **9** (DMSO-d_6, 500 MHz). 18

Fig. S18 ^{13}C NMR spectrum of compound **9** (DMSO-d_6, 125 MHz). 18

Fig. S19 ^{1}H NMR spectrum of compound **10** (DMSO-d_6, 500 MHz) 19

Fig. S20 ^{13}C NMR spectrum of compound **10** (DMSO-d_6, 125 MHz). 19

Fig. S21 ^{1}H NMR spectrum of compound **11** (DMSO-d_6, 500 MHz). 20

Fig. S22 ^{13}C NMR spectrum of compound **11** (DMSO-d_6, 125 MHz). 20

Fig. S23 ^{1}H NMR spectrum of compound **12** (DMSO-d_6, 500 MHz). 21
Fig. S24 ^{13}C NMR spectrum of compound **12** (DMSO-d_6, 125 MHz). 21
Fig. 25 ^{1}H NMR spectrum of compound **13** (DMSO-d_6, 400 MHz)......................................22
Fig. 26 ^{13}C NMR spectrum of compound **13** (DMSO-d_6, 100 MHz)....................................22
Fig. S27 Dose response (cytotoxicity) curve of the total extract and different fractions against HCT-116 (A), MCF-7 (B) and HepG-2 (C) cell lines. 23
Fig. S28 Dose response (cytotoxicity) curve of some isolated compounds from *Dypsis pembana* leaves against HepG-2 cells. 23
Fig. S29 2D (A) and 3D (B) interactions of compound **11** with topoisomerase IIα (PDB ID: 5gwk). 23
Fig. S30 2D (A) and 3D (B) interactions of compound **12** with topoisomerase IIα (PDB ID: 5gwk). 24
Fig. S31 2D (A) and 3D (B) interactions of compound **13** with topoisomerase IIα (PDB ID: 5gwk). 24
Fig. S32 2D (A) and 3D (B) interactions of compound **11** with cyclin-dependent kinase 2 (PDB ID: 3ti1)..24
Fig. S33 2D (A) and 3D (B) interactions of compound **12** with cyclin-dependent kinase 2 (PDB ID: 3ti1)..25
Fig S34 2D (A) and 3D (B) interactions of compound **13** with cyclin-dependent kinase 2 (PDB ID: 3ti1)..25

List of Tables

Table S1 Binding scores of the docking on topoisomerase IIα (PDB ID: 5gwk) and cyclin-dependent kinase 2 (PDB ID: 3ti1)

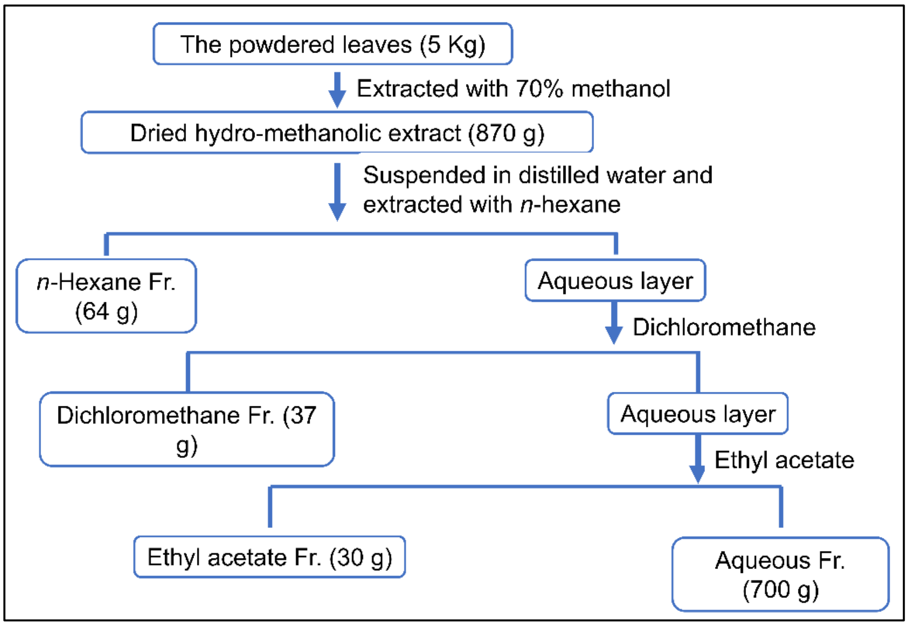

Scheme S1: Extraction and fractionation of *Dypsis pembana* leaves

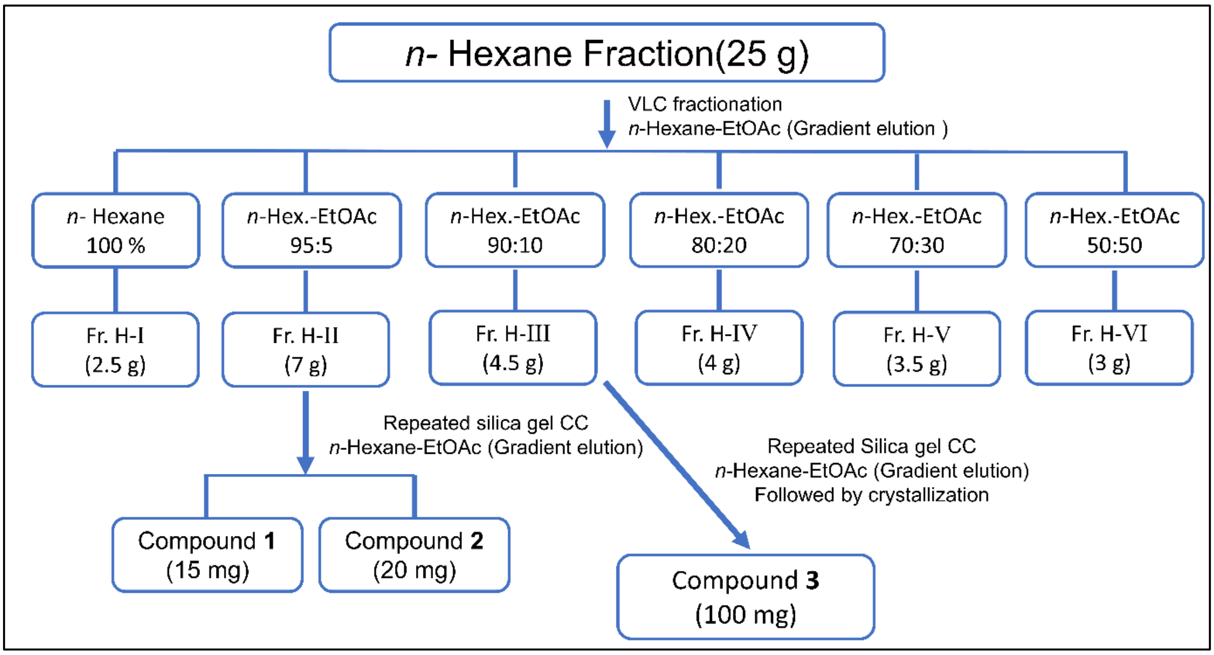

Scheme S2: Isolation and purification of compounds **1**–**3** from *n*-hexane fraction of *Dypsis pembana* leaves

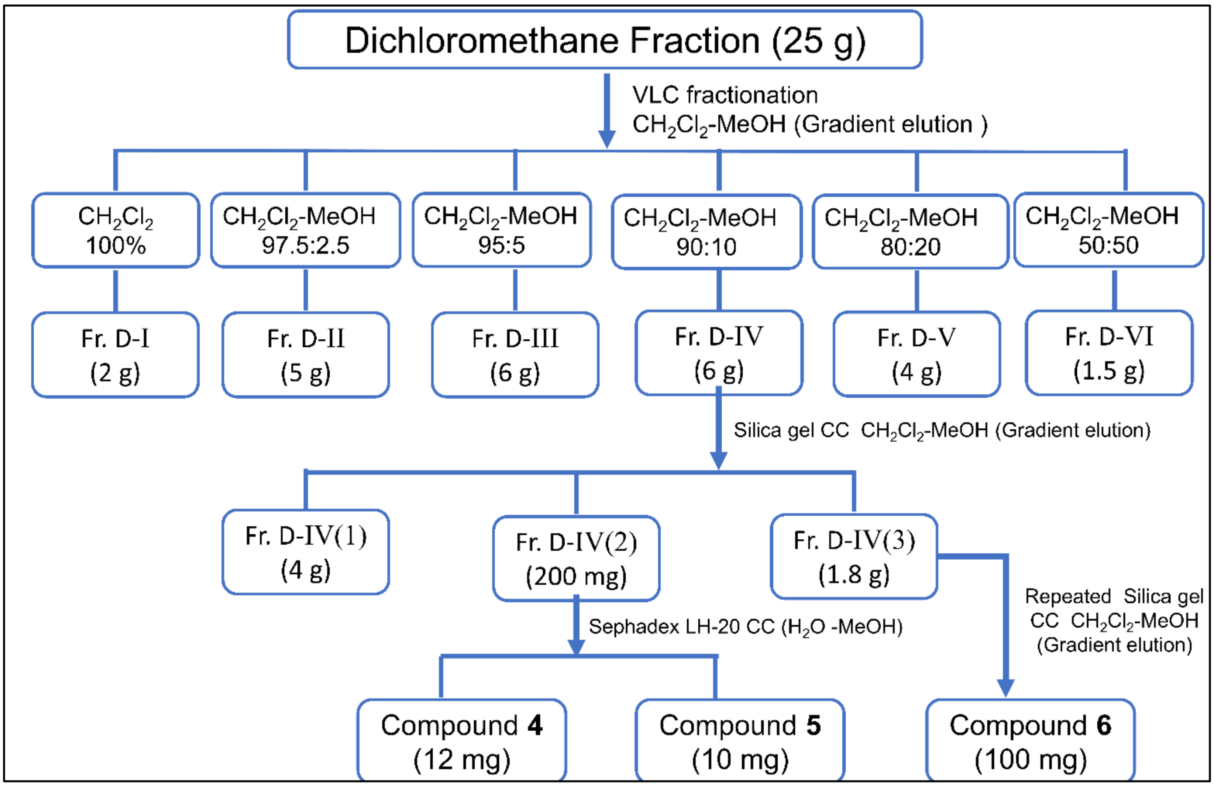

Scheme S3: Isolation and purification of compounds **4**–**6** from the dichloromethane fraction of *Dypsis pembana* leaves

S 1 Physicochemical and spectral data of the isolated compounds (**1**–**13**)

Compound 1 was obtained from *n*-hexane fraction as colourless needles (15 mg). ^{1}H-NMR ($CDCl_3$, 400 MHz): 5.263 (1H,*d*, *J* = 6.0 Hz, H-11), 3.425 (1H *t, J* = 2.8 Hz, H-3), 0.956 (3H, *s,* H_3-23), 0.876 (3H, *s,* H_3-24), 1.051 (3H, *s,* H_3-25), 0.819 (3H, *s,* H_3-26), 0.771 (3H, *s,* H_3-27), 0.755 (3H, *s,* H_3-28), 0.890 (3H*, d*, *J* = 6.4 Hz, H_3-29), 0.828 (3H*, d*, *J* = 6.6 Hz, H_3-30). DEPTQ ($CDCl_3$, 100 MHz): 30.40 (C-1), 25.69 (C-2), 76.28 (C-3), 37.84 (C-4), 46.55 (C-5), 21.36 (C-6), 26.62 (C-7), 41.03 (C-8), 148.81 (C-9), 39.60 (C-10), 114.08 (C-11), 36.05 (C-12), 36.76 (C-13), 38.27 (C-14), 29.63 (C-15), 35.94 (C-16), 42.87 (C-17), 52.09 (C-18), 20.19 (C-19), 28.23 (C-20), 59.64 (C-21), 30.80 (C-22), 28.30 (C-23), 22.53 (C-24), 21.93 (C-25), 17.06 (C-26), 15.30 (C-27), 14.01 (C-28), 22.13 (C-29), 23.01 (C-30).

Compound 2 was obtained from *n*-hexane fraction as white amorphous powder (20 mg). ^{1}H-NMR ($CDCl_3$, 400 MHz): 5.228 (1H,*d*, *J* = 6.1 Hz, H-11), 3.214 (1H *dd, J* = 11.5, 4.3 Hz, H-3), 0.984 (3H, *s,* H_3-23), 0.815 (3H, *s,* H_3-24), 1.032 (3H, *s,* H_3-25), 0.805 (3H, *s,* H_3-26), 0.768 (3H, *s,* H_3-27), 0.756 (3H, *s,* H_3-28), 0.890 (3H*, d*, *J* = 6.4 Hz, H_3-29), 0.828 (3H*, d*, *J* = 6.4 Hz, H_3-30). DEPTQ ($CDCl_3$, 100 MHz): 36.20 (C-1), 27.95 (C-2), 79.12 (C-3), 39.78 (C-4), 52.49 (C-5), 21.57 (C-6), 26.83 (C-7), 41.11 (C-8), 149.02 (C-9), 39.22 (C-10), 114.48 (C-11), 36.15 (C-12), 36.92 (C-13), 38.33 (C-14), 29.85 (C-15), 36.07 (C-16), 43.00 (C-17), 52.23 (C-18), 20.32 (C-19), 28.36 (C-20), 59.80 (C-21), 30.93 (C-22), 28.37 (C-23), 15.78 (C-24), 22.27 (C-25), 17.18 (C-26), 15.43 (C-27), 14.14 (C-28), 22.27 (C-29), 23.15 (C-30).

Compound 3 was obtained from *n*-hexane fraction as white crystalline needles (100 mg). ^{1}H-NMR ($CDCl_3$, 400 MHz): 5.336 (2H,*d*, *J* = 5.2 Hz, H-6, H-6`), 5.142 (1H, *dd*, *J* = 8.6, 15.1 Hz, H-22`), 5.001 (1H, *dd*, *J* = 8.6, 15.1 Hz, H-22`), 3.509 (2H, *m*, H-3, H-3`), 0.670 (6H, *s,* H_3-18, H_3-18`), 0.997 (6H, *s,* $H_3$-19, $H_3$-19`), 0.913 (6H, *d*, *J* = 6.4 Hz*,* H_3-21, H_3-21`), 0.827 (6H, *d*, *J* = 6.8 Hz*,* $H_3$- 26, $H_3$-26`), 0.805 (6H, *d*, *J* = 6.8 Hz*,* H_3-27, H_3-27`), 0.790-0.850 (6H, *overlapped,* $H_3$-29, $H_3$-29). [13]C-NMR ($CDCl_3$, 100 MHz): 37.39 (C-1), 37.39 (C-1`), 31.75 (C-2), 31.75 (C-2`), 71.89 (C-3), 71.89 (C-3`), 42.45 (C-4), 42.45 (C-4`), 140.89 (C-5), 140.89 (C-5`), 121.81 (C-6), 121.81 (C-6`), 32.04 (C-7), 32.04 (C-7`), 32.04 (C-8), 32.04 (C-8`), 50.27 (C-9), 50.27 (C-9`), 36.63 (C-10), 36.63 (C-10`), 21.22 (C-11), 21.22 (C-11`), 39.91 (C-12), 39.82 (C-12`), 42.40 (C-13), 42.40 (C-13`), 56.90 (C-14), 57.00 (C-14`), 24.43 (C-15), 24.49 (C-15`), 28.37 (C-16), 29.04 (C-16`), 56.20 (C-17), 56.09 (C-17`), 12.11 (C-18), 12.18 (C-18`), 19.52 (C-19), 19.52 (C-19`), 36.27 (C-20), 40.62 (C-20`), 18.84 (C-21), 21.22 (C-21`), 34.08 (C-22), 138.44 (C-22`), 26.23 (C-23), 129.41 (C-23`), 45.97 (C-24), 51.37 (C-

24`), 29.30 (C-25), 32.04 (C-25`), 19.18 (C-26), 21.35 (C-26`), 19.59 (C-27), 18.92 (C-27`), 23.20 (C-28), 25.53 (C-28`), 11.99 (C-29), 12.38 (C-29`)

Compound 4 was isolated from dichloromethane fraction as yellow amorphous powder (12 mg). ^{1}H-NMR (DMSO-d_6, 400 MHz): 12.471(1H, *s,* OH-5), 8.037 (2H, *d*, *J* = 8.8 Hz, H-2',6'), 6.923 (2H *d, J* = 8.8 Hz, H-3',5'), 6.430 (1H, *brs,* H-8), 6.182 (1H, *brs,* H-6). ^{13}C-NMR (DMSO-d_6, 100 MHz): 146.81 (C-2), 135.68 (C-3), 175.92 (C-4), 160.71 (C-5), 98.21 (C-6), 163.92 (C-7), 93.49 (C-8), 156.18 (C-9), 103.03 (C-10), 121.67 (C-1'), 129.51 (C-2',6'), 115.45 (C-3',5'), 159.20 (C-4').

Compound 5 was isolated from dichloromethane fraction as yellow amorphous powder (10 mg). ^{1}H-NMR (DMSO-d_6, 400 MHz): 12.462 (1H, *s,* OH-5), 7.664 (1H, *d*, *J* = 2.2 Hz, H-2'), 7.536 (1H, *dd*, *J* = 8.6, 2.2 Hz, H-6'), 6.877 (1H, *d*, *J* = 8.4 Hz, H-5'), 6.401 (1H, *d*, *J* = 2.2 Hz, H-8), 6.177 (1H, *d*, *J* = 2.2 Hz, H-6). ^{13}C-NMR (DMSO-d_6, 100 MHz): 146.80 (C-2), 134.91 (C-3), 175.88 (C-4), 160.74 (C-5), 98.24 (C-6), 164.01 (C-7), 93.40 (C-8), 156.17 (C-9), 103.00 (C-10), 122.00 (C-1'), 115.63 (C-2'), 145.10 (C-3'), 147.74 (C-4'), 116.59 (C-5'), 120.00 (C-6')

Compound 6 was obtained from dichloromethane fraction as white amorphous powder (100 mg). ^{1}H-NMR (DMSO-d_6, 400 MHz): 5.323 (1H, *brs*, H-6), 4.411 (1H, *brs*, H-3), 0.648 (3H, *s,* H_3-18), 0.953 (3H, *s,* H_3-19), 0.897 (3H, *d*, *J* = 6.6 Hz, H_3-21), 0.809 (6H, *d*, *J* = 7.2 Hz, H_3-26), 0.750-0.830 (6H, *overlapped,* H_3-27, 29), 4.213 (1H, *d*, *J* = 7.7 Hz, H-1'), 2.850-3.690 (Sugar protons, *m*). ^{13}C-NMR (DMSO-d_6, 100 MHz): 36.83 (C-1), 29.26 (C-2), 76.96 (C-3), 41.84 (C-4), 140.44 (C-5), 121.16 (C-6), 31.37 (C-7), 31.41 (C-8), 49.61 (C-9), 36.20 (C-10), 20.60 (C-11), 38.30 (C-12), 41.85 (C-13), 56.18 (C-14), 23.86 (C-15), 27.78 (C-16), 55.45 (C-17), 11.65 (C-18), 19.68 (C-19), 35.49 (C-20), 19.08 (C-21), 33.35 (C-22), 25.46 (C-23), 45.15 (C-24), 28.71 (C-25), 18.93 (C-26), 18.60 (C-27), 22.61 (C-28), 11.77 (C-29), 100.83 (C-1'), 73.45 (C-2'), 76.76 (C-3'), 70.07 (C-4'), 76.72 (C-5'), 61.07 (C-6')

Compound 7 was isolated from ethyl acetate fraction as yellow amorphous powder (8 mg). ^{1}H-NMR (DMSO-d_6, 400 MHz): 13.616 (1H, *s,* OH-5), 7.886 (2H,*d*, *J* = 8.8 Hz, H-2',6'), 6.915 (2H *d, J* = 8.8 Hz, H-3',5'), 6.691 (1H, *s,* H-3), 4.701 (1H, *d*, *J* = 9.7 Hz, H-1'''), 4.581 (1H, *d*, *J* = 9.8 Hz, H-1''), 2.950-3.850 (Sugars protons, *m*). ^{13}C-NMR (DMSO-d_6, 100 MHz): 163.21 (C-2), 100.85 (C-3), 181.29 (C-4), 159.34 (C-5), 109.08 (C-6), 162.47 (C-7), 105.06 (C-8), 156.48 (C-9), 102.62 (C-10), 121.12 (C-1'), 128.38 (C-2', 6'), 116.10 (C-3', 5'), 161.36 (C-4'), 73.30 (C-1''), 70.57 (C-2''), 79.09 (C-3''), 70.06 (C-4''), 81.39 (C-5''), 61.42 (C-6''), 73.93 (C-1'''), 70.97 (C-2'''), 79.48 (C-3'''), 70.22 (C-4'''), 81.49 (C-5'''), 61.69 (C-6''')

Compound 8 was isolated from ethyl acetate fraction as yellow amorphous powder (20 mg). ^{1}H-NMR (DMSO-d_6, 500 MHz): 12.586 (1H, *s,* OH-5), 7.548 (1H, *d*, J = 2.0 Hz, H-2'), 7.527 (1H, *overlapped,* H-6'), 6.836 (1H *d,* J = 8.0 Hz, H-5'), 6.372 (1H, *d*, J = 2.0 Hz, H-8), 6.179 (1H, *d*, J = 2.0 Hz, H-6), 5.334 (1H, *d*, J = 7.5 Hz, H-1''), 4.382 (1H, *brs*, H-1'''), 3.032-3.790 (Sugars protons, *m*), 0.992 (3H, *d*, J = 6.5 Hz, H-6'''). ^{13}C-NMR (DMSO-d_6, 125 MHz): 156.46 (C-2), 133.30 (C-3), 177.33 (C-4), 161.22 (C-5), 98.77 (C-6), 164.42 (C-7), 93.64 (C-8), 156.57 (C-9), 103.86 (C-10), 121.15 (C-1'), 116.25 (C-2'), 144.79 (C-3'), 148.49 (C-4'), 115.25 (C-5'), 121.60 (C-6'), 101.24 (C-1''), 74.09 (C-2''), 75.91 (C-3''), 70.01 (C-4''), 76.47 (C-5''), 67.00 (C-6''), 100.75 (C-1'''), 70.37 (C-2'''), 70.57 (C-3'''), 71.86 (C-4'''), 68.24 (C-5'''), 17.73 (C-6''')

Compound 9 was isolated from ethyl acetate fraction as yellow amorphous powder (10 mg). ^{1}H-NMR (DMSO-d_6, 500 MHz): 12.626 (1H, *s,* OH-5), 8.015 (2H, *d*, J = 9.0 Hz, H-2', 6'), 6.868 (2H *d,* J = 9.0 Hz, H-3',5'), 6.335 (1H, *brs,* H-8), 6.118 (1H, *brs,* H-6), 5.662 (1H, *d*, J = 7.5 Hz, H-1''), 5.070 (1H, *brs*, H-1'''), 3.070-3.950 (Sugars protons, *m*), 0.761 (3H, *d*, J = 6.3 Hz, H-6'''). ^{13}C-NMR (DMSO-d_6, 125 MHz): 155.67 (C-2), 132.56 (C-3), 177.06 (C-4), 161.20 (C-5), 98.33 (C-6), 166.54 (C-7), 93.95 (C-8), 156.57 (C-9), 103.20 (C-10), 120.98 (C-1'), 130.69 (C-2', 6'), 115.15 (C-3', 5'), 160.00 (C-4'), 100.60 (C-1''), 77.44 (C-2''), 77.33 (C-3''), 70.65 (C-4''), 77.61 (C-5''), 60.81 (C-6''), 99.35 (C-1'''), 70.59 (C-2'''), 70.26 (C-3'''), 71.89 (C-4'''), 68.33 (C-5'''), 17.26 (C-6''')

Compound 10 was isolated from ethyl acetate fraction as yellow amorphous powder (12 mg). ^{1}H-NMR (DMSO-d_6, 500 MHz): 12.631 (1H, *s,* OH-5), 7.583 (1H, *d*, J = 2.5 Hz, H-2'), 7.568 (1H, *overlapped,* H-6'), 6.836 (1H *d,* J = 9.0 Hz, H-5'), 6.391 (1H, *brs,* H-8), 6.190 (1H, *brs,* H-6), 5.458 (1H, *d*, J = 7.5 Hz, H-1''), 3.070-3.690 (Sugar protons, *m*). ^{13}C-NMR (DMSO-d_6, 125 MHz): 156.16 (C-2), 133.32 (C-3), 177.43 (C-4), 161.24 (C-5), 98.70 (C-6), 164.27 (C-7), 93.52 (C-8), 156.34 (C-9), 103.93 (C-10), 121.16 (C-1'), 115.22 (C-2'), 144.82 (C-3'), 148.49 (C-4'), 116.19 (C-5'), 121.60 (C-6'), 100.89 (C-1''), 74.10 (C-2''), 76.51 (C-3''), 69.94 (C-4''), 77.56 (C-5''), 60.98 (C-6'')

Compound 11 was isolated from ethyl acetate fraction as yellow amorphous powder (22 mg). ^{1}H-NMR (DMSO-d_6, 500 MHz): 13.176 (1H, *s,* OH-5), 7.512 (1H, *overlapped*, H-2'), 7.466 (1H, *overlapped,* H-6'), 6.856 (1H *d,* J = 8.5 Hz, H-5'), 6.624 (1H, *s,* H-3), 6.246 (1H, *s,* H-6), 4.691 (1H, *d*, J = 10.0 Hz, H-1''), 3.050-3.910 (Sugar protons, *m*). ^{13}C-NMR (DMSO-d_6, 125 MHz): 164.04 (C-2), 102.29 (C-3), 181.92 (C-4), 160.41 (C-5), 98.28 (C-6), 163.13 (C-7), 104.57 (C-8), 156.01 (C-9), 103.80 (C-10), 121.77 (C-1'), 113.92 (C-2'), 145.92 (C-3'), 149.96

(C-4'), 115.73 (C-5'), 119.33 (C-6'), 73.42 (C-1''), 70.84 (C-2''), 78.78 (C-3''), 70.73 (C-4''), 81.98 (C-5''), 61.67 (C-6'')

Compound 12 was isolated from ethyl acetate fraction as yellow amorphous powder (20 mg). ^{1}H-NMR (DMSO-d_6, 500 MHz): 13.157 (1H, *s,* OH-5), 8.016 (2H *d, J* = 8.5 Hz, H-2',6'), 6.897 (2H, *d, J* = 8.5 Hz, H-3',5'), 6.751 (1H, *s,* H-3), 6.257 (1H, *s,* H-6), 4.720 (1H, *d*, *J* = 9.5 Hz, H-1''), 3.150-3.920 (Sugar protons, *m*). ^{13}C-NMR (DMSO-d_6, 125 MHz): 163.87 (C-2), 102.40 (C-3), 181.99 (C-4), 161.34 (C-5), 98.50 (C-6), 161.34 (C-7), 104.71 (C-8), 156.10 (C-9), 103.72 (C-10), 121.61 (C-1'), 128.96 (C-2', 6'), 115.94 (C-3', 5'), 160.48 (C-4'), 73.48 (C-1''), 70.96 (C-2''), 78.77 (C-3''), 70.65 (C-4''), 81.84 (C-5''), 61.38 (C-6'').

Compound 13 was isolated from ethyl acetate fraction as yellow amorphous powder (15 mg). ^{1}H-NMR (DMSO-d_6, 400 MHz): 13.620 (1H, *s,* OH-5), 7.897 (2H *d, J* = 9.0 Hz, H-2',6'), 6.917 (2H, *d, J* = 9.0 Hz, H-3',5'), 6.720 (1H, *s,* H-3), 6.461 (1H, *s,* H-8), 4.595 (1H, *d*, *J* = 10.0 Hz, H-1''), 3.050-3.920 (Sugar protons, *m*). ^{13}C-NMR (DMSO-d_6, 100 MHz): 163.27 (C-2), 102.61 (C-3), 181.69 (C-4), 161.42 (C-5), 109.04 (C-6), 160.70 (C-7), 93.95 (C-8), 156.42 (C-9), 105.03 (C-10), 121.03 (C-1'), 128.39 (C-2', 6'), 116.09 (C-3', 5'), 160.33 (C-4'), 73.25 (C-1''), 70.59 (C-2''), 79.08 (C-3''), 70.19 (C-4''), 81.51 (C-5''), 61.44 (C-6'')

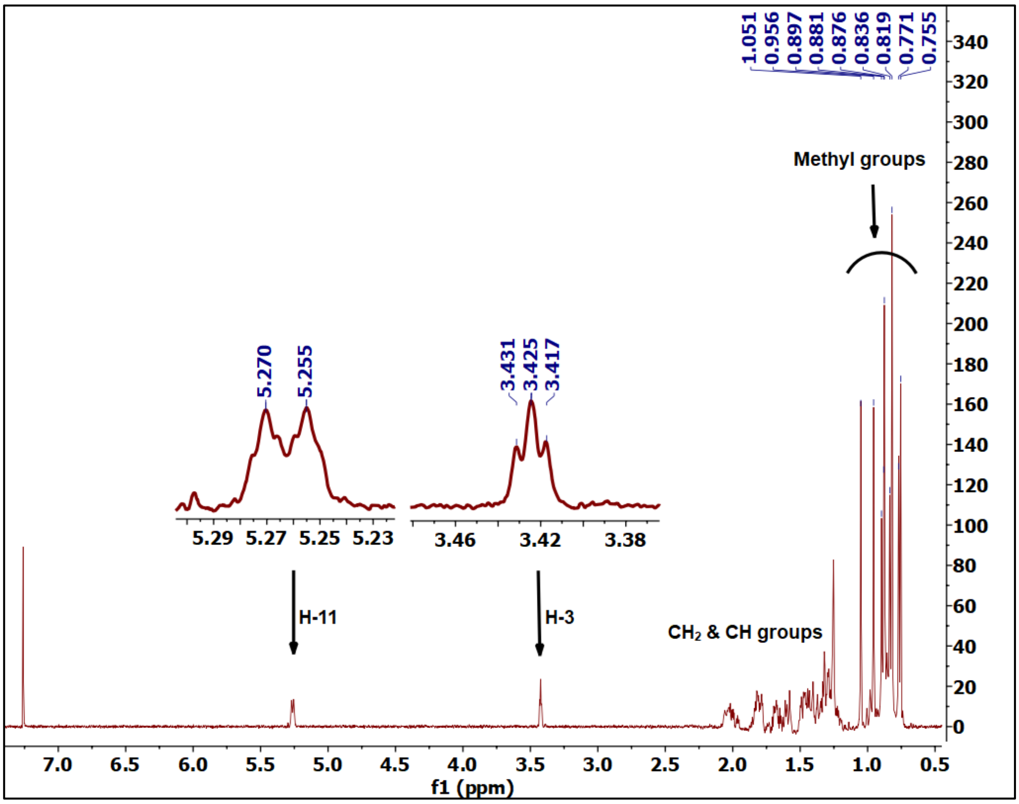

Fig. S1 ^{1}H NMR spectrum of compound **1** ($CDCl_3$, 400 MHz).

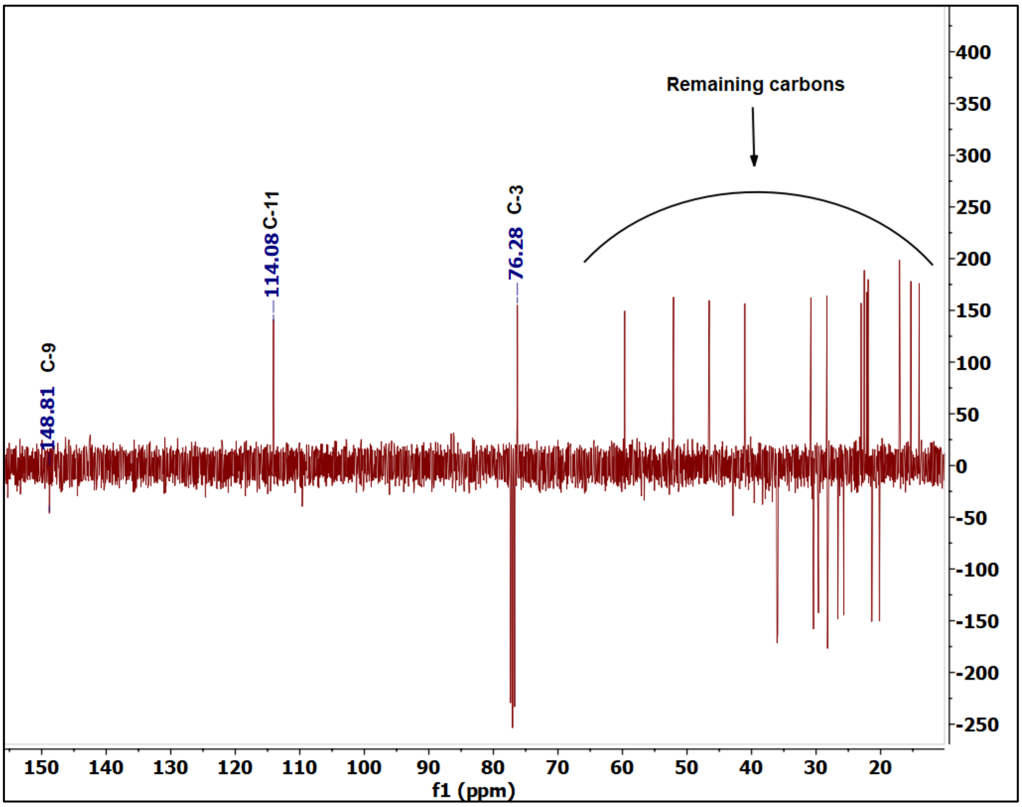

Fig. S2 DEPT-Q spectrum of compound **1** ($CDCl_3$, 100 MHz).

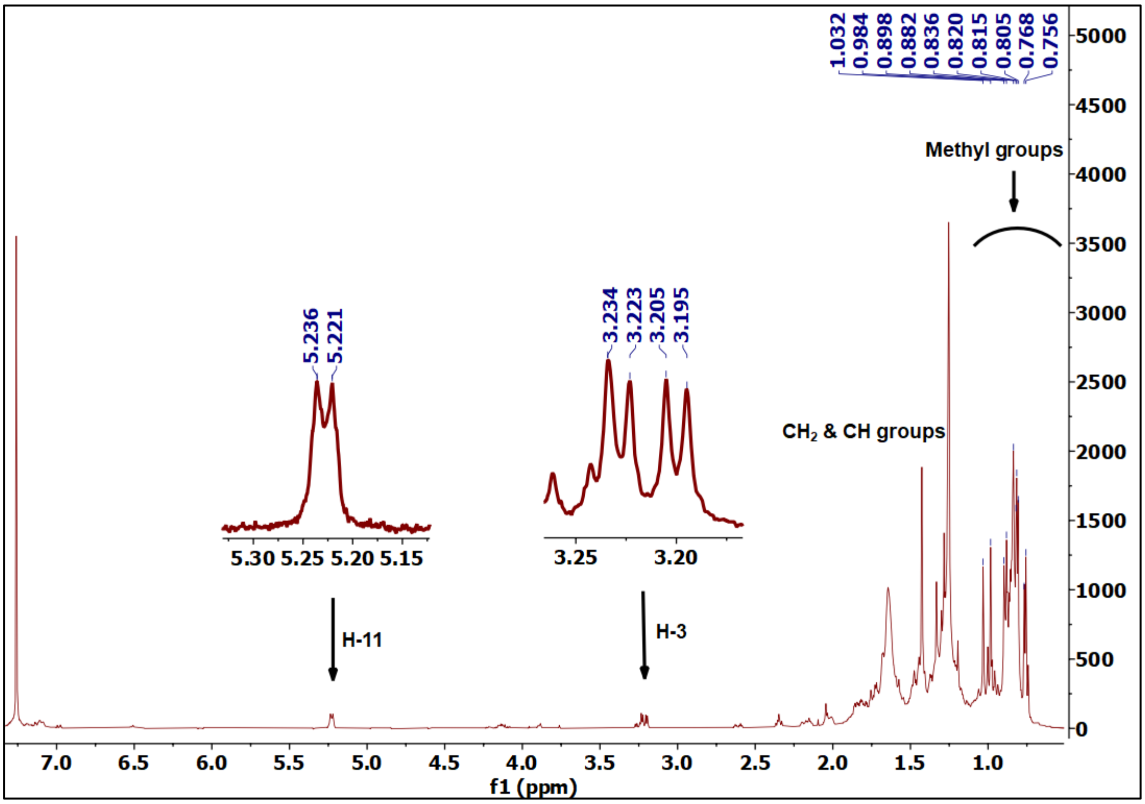

Fig. S3 ^{1}H NMR spectrum of compound **2** ($CDCl_3$, 400 MHz).

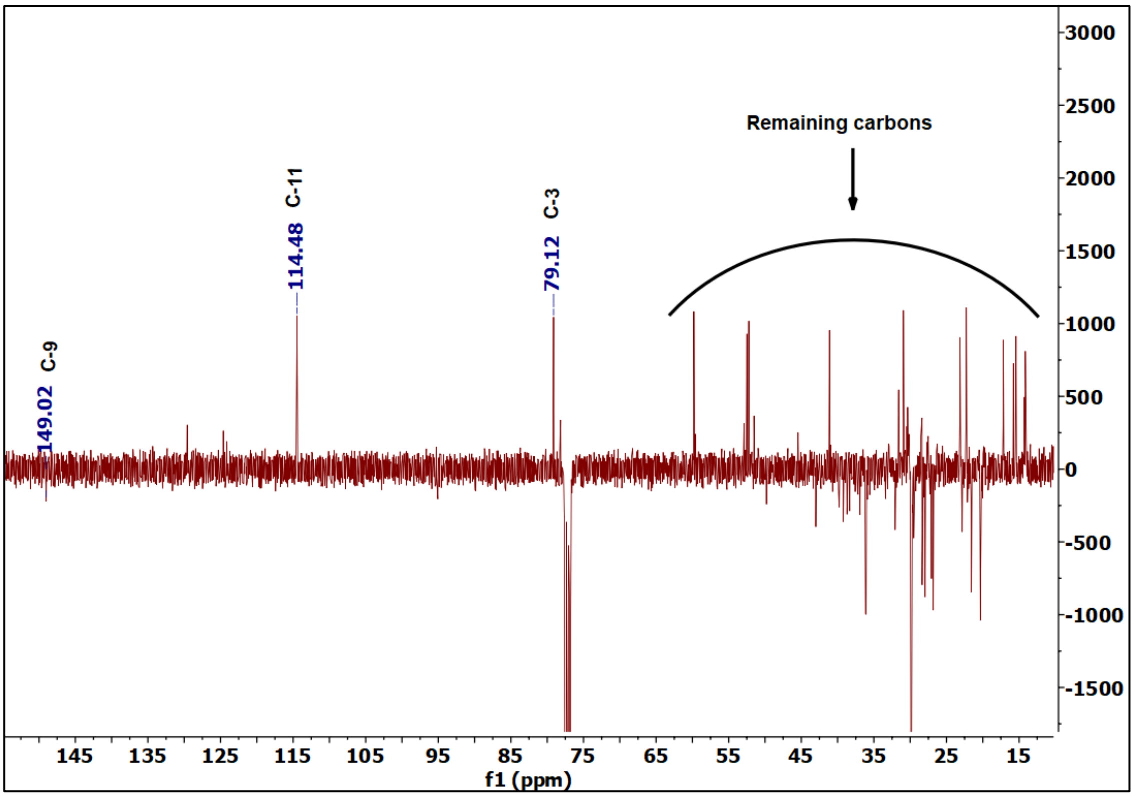

Fig. S4 DEPT-Q spectrum of compound **2** ($CDCl_3$, 100 MHz).

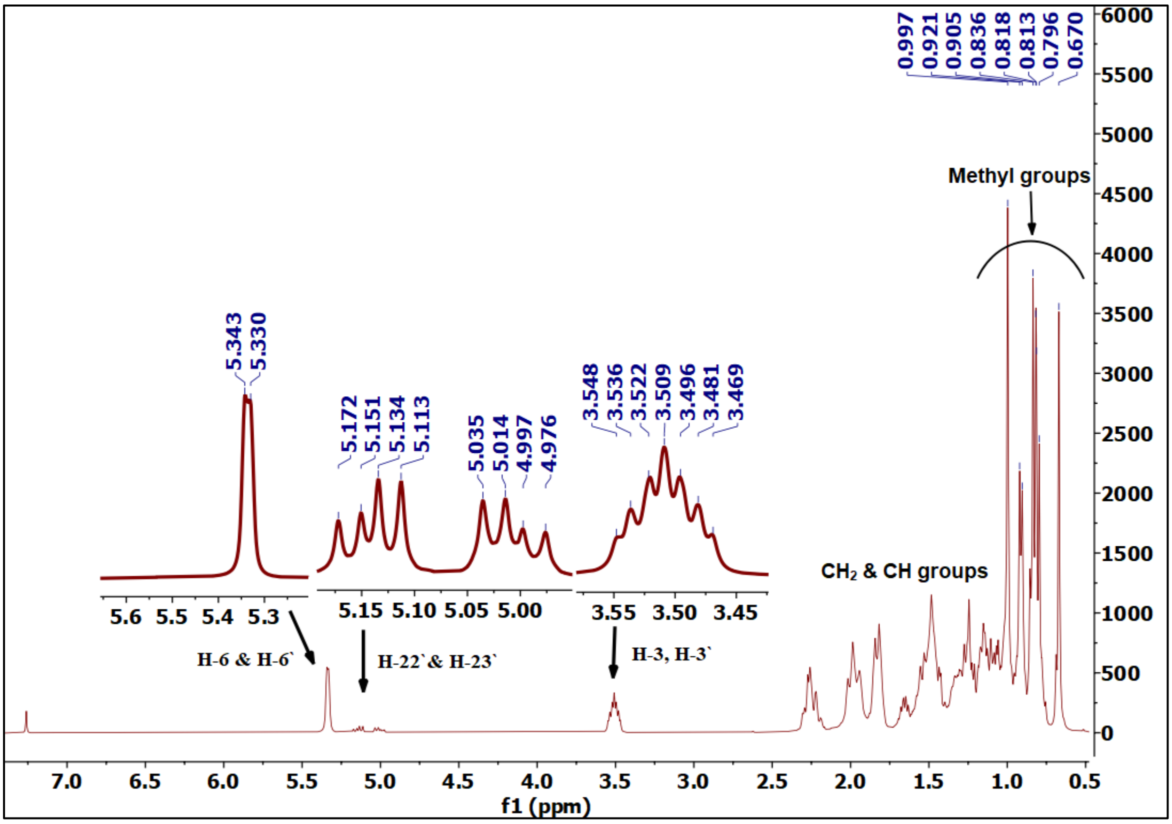

Fig. S5 ^{1}H NMR spectrum of compound **3** ($CDCl_3$, 400 MHz).

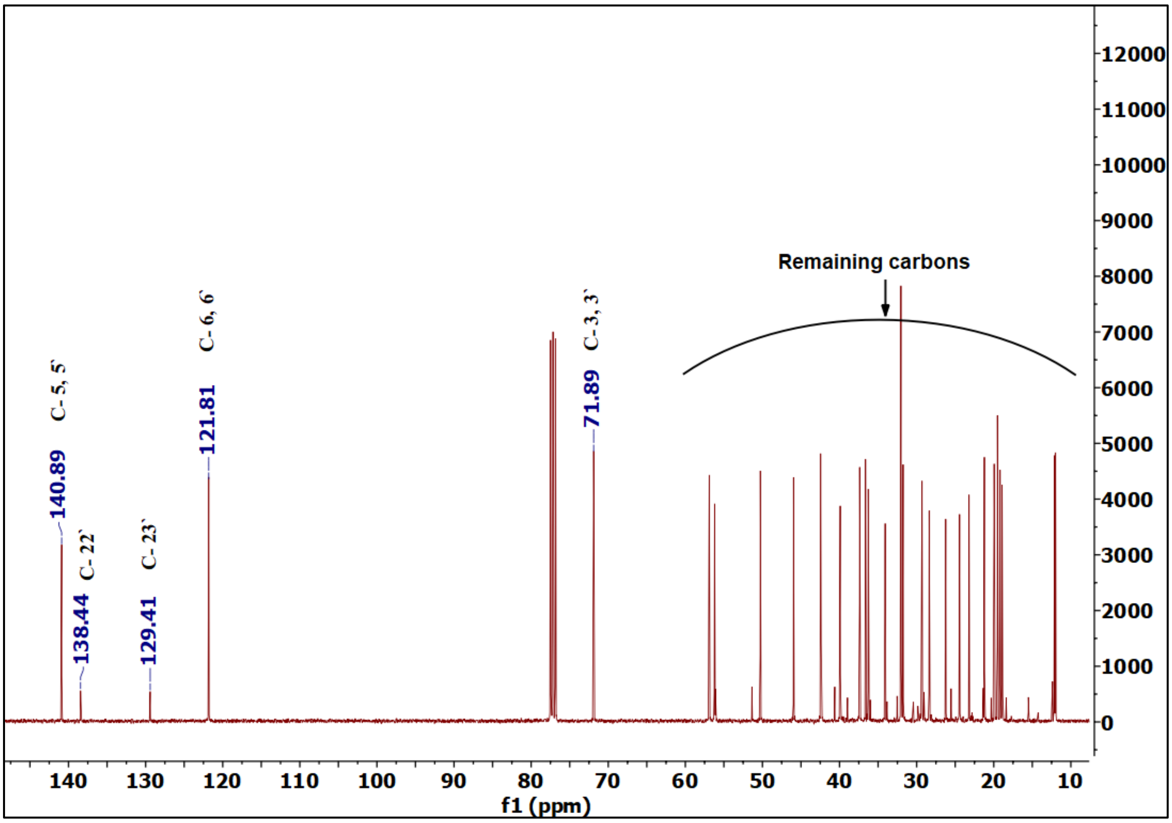

Fig. S6 ^{13}C NMR spectrum of compound **3** ($CDCl_3$, 100 MHz).

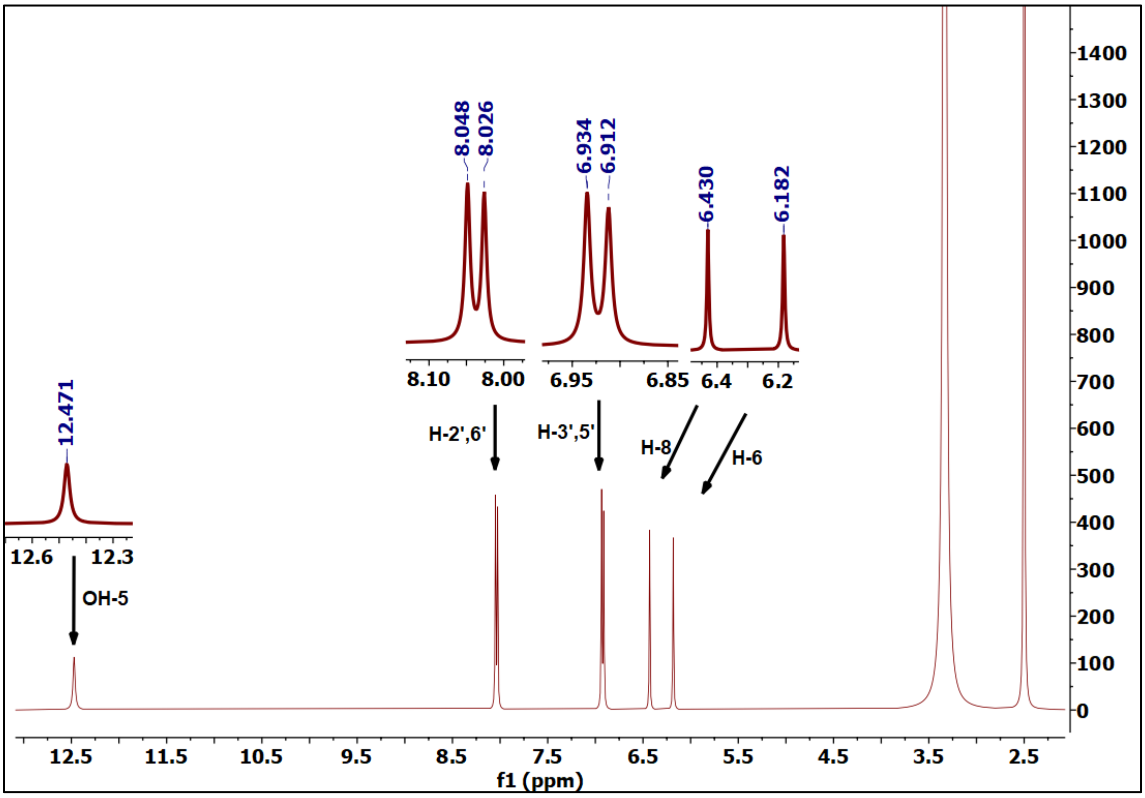

Fig. S7 ^{1}H NMR spectrum of compound **4** (DMSO-d_6, 400 MHz).

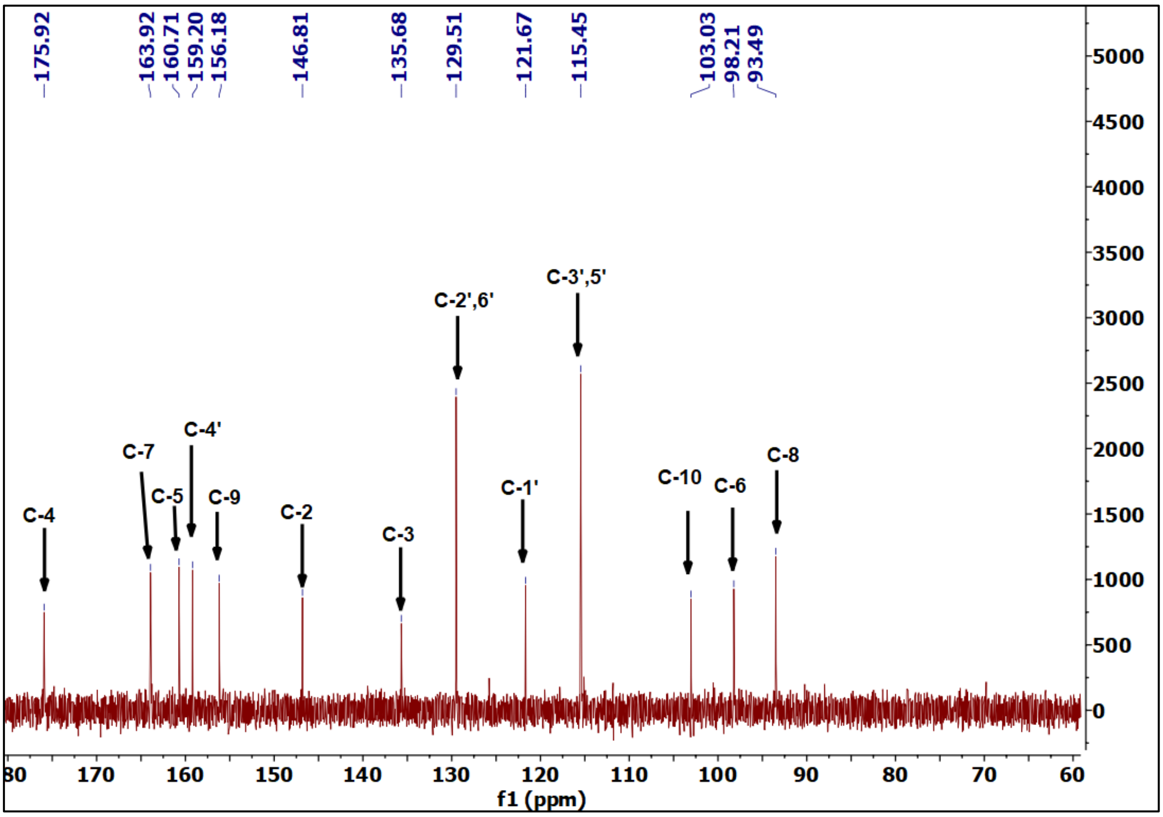

Fig. S8 ^{13}C NMR spectrum of compound **4** (DMSO-d_6, 100 MHz).

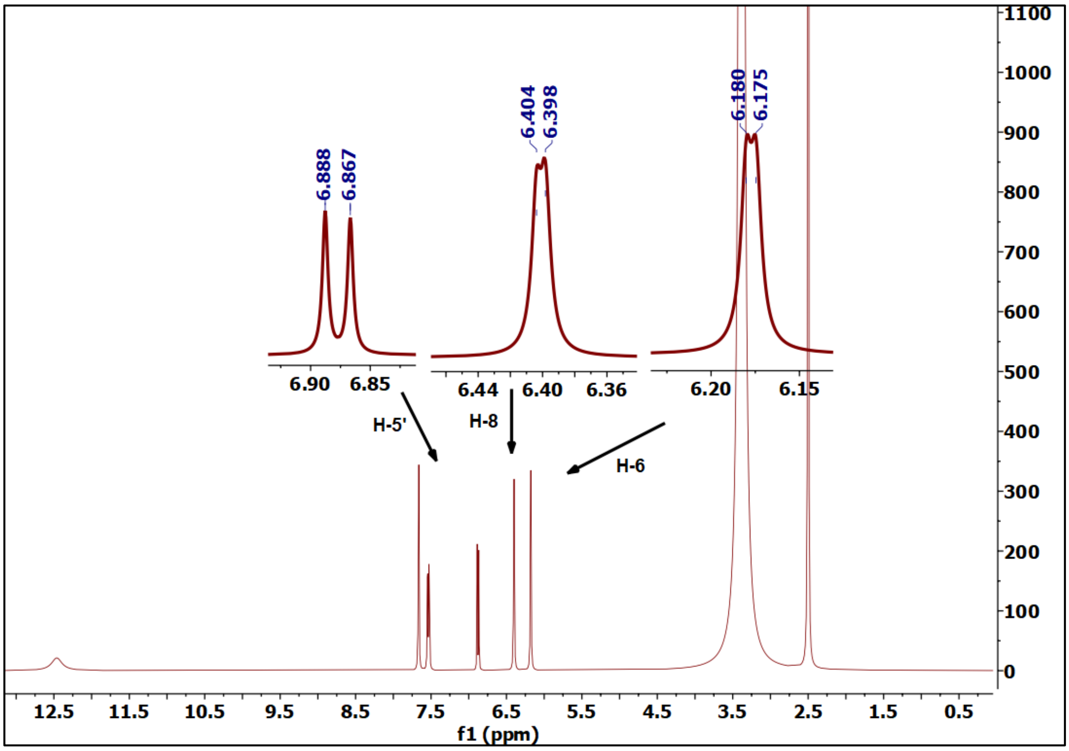

Fig. S9 ^{1}H NMR spectrum of compound **5** (DMSO-d_6, 400 MHz).

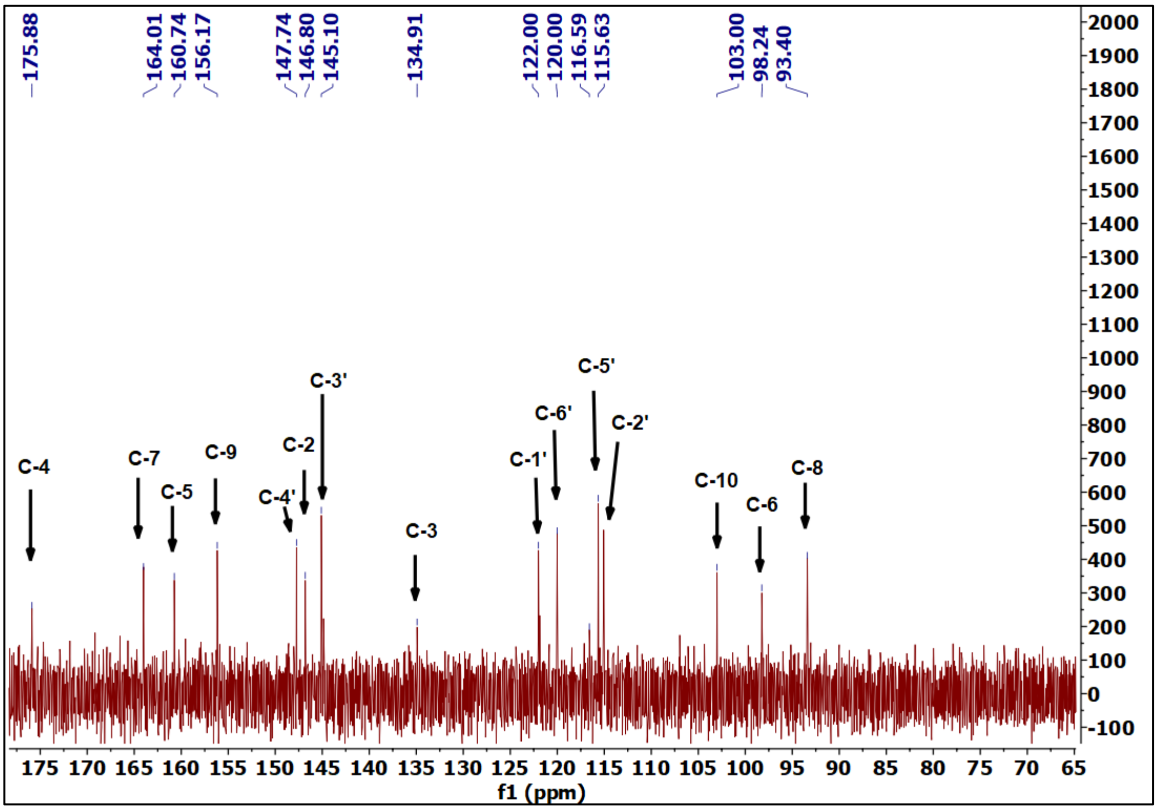

Fig. S10 ^{13}C NMR spectrum of compound **5** (DMSO-d_6, 100 MHz).

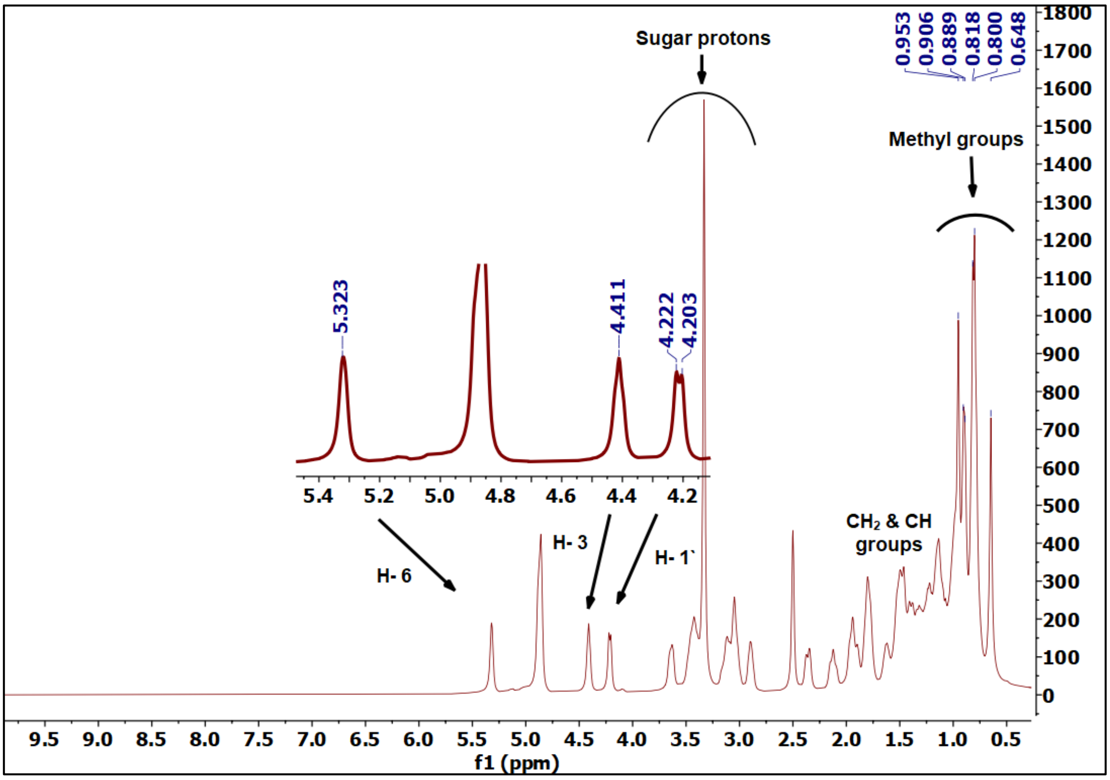

Fig. S11 ^{1}H NMR spectrum of compound **6** (DMSO-d_6, 400 MHz).

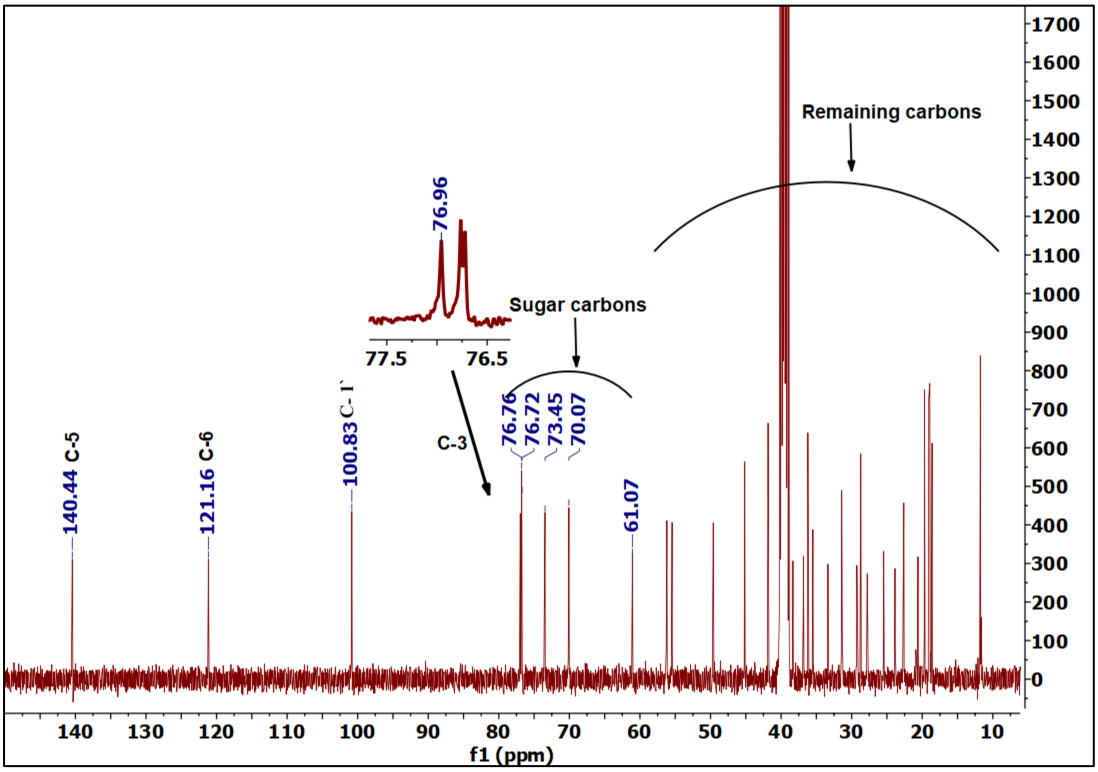

Fig. S12 ^{13}C NMR spectrum of compound **6** (DMSO-d_6, 100 MHz).

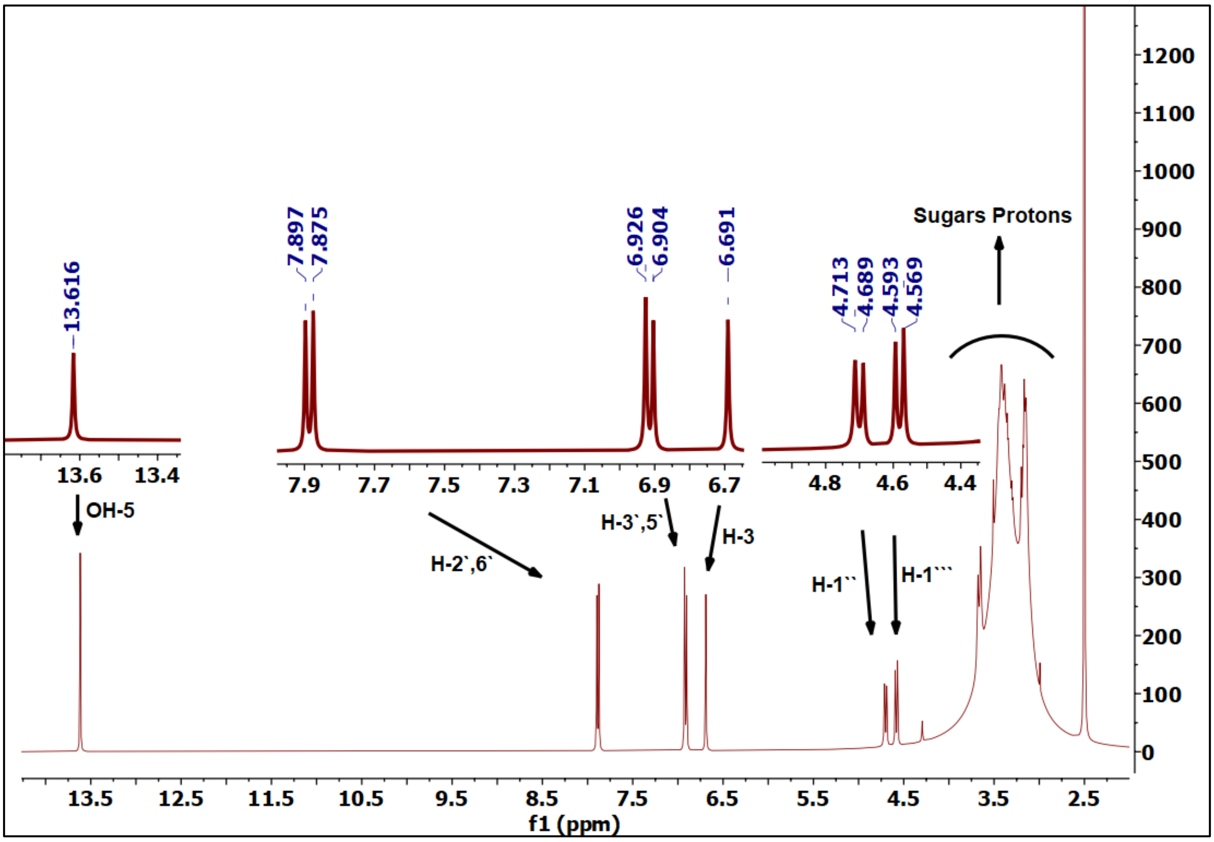

Fig. S13 ^{1}H NMR spectrum of compound **7** (DMSO-d_6, 400 MHz).

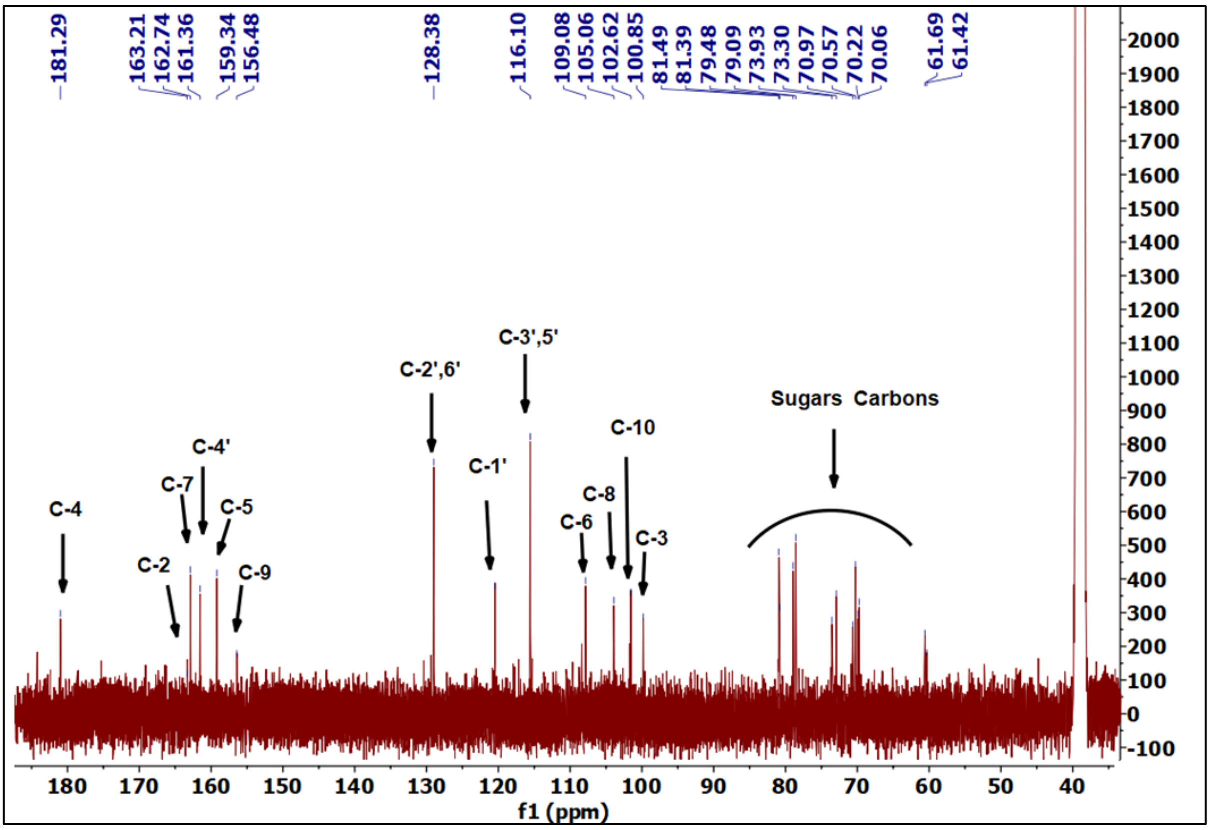

Fig. S14 ^{13}C NMR spectrum of compound **7** (DMSO-d_6, 100 MHz).

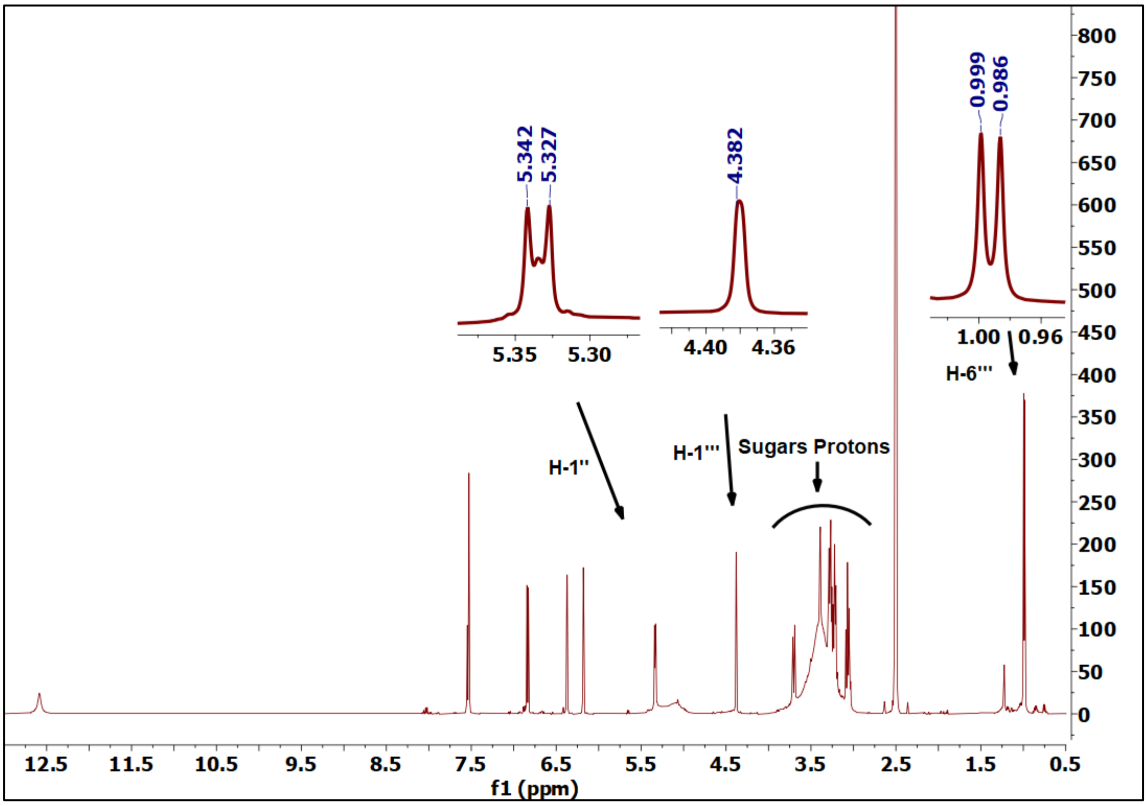

Fig. S16 ^{1}H NMR spectrum of compound **8** (DMSO-d_6, 500 MHz).

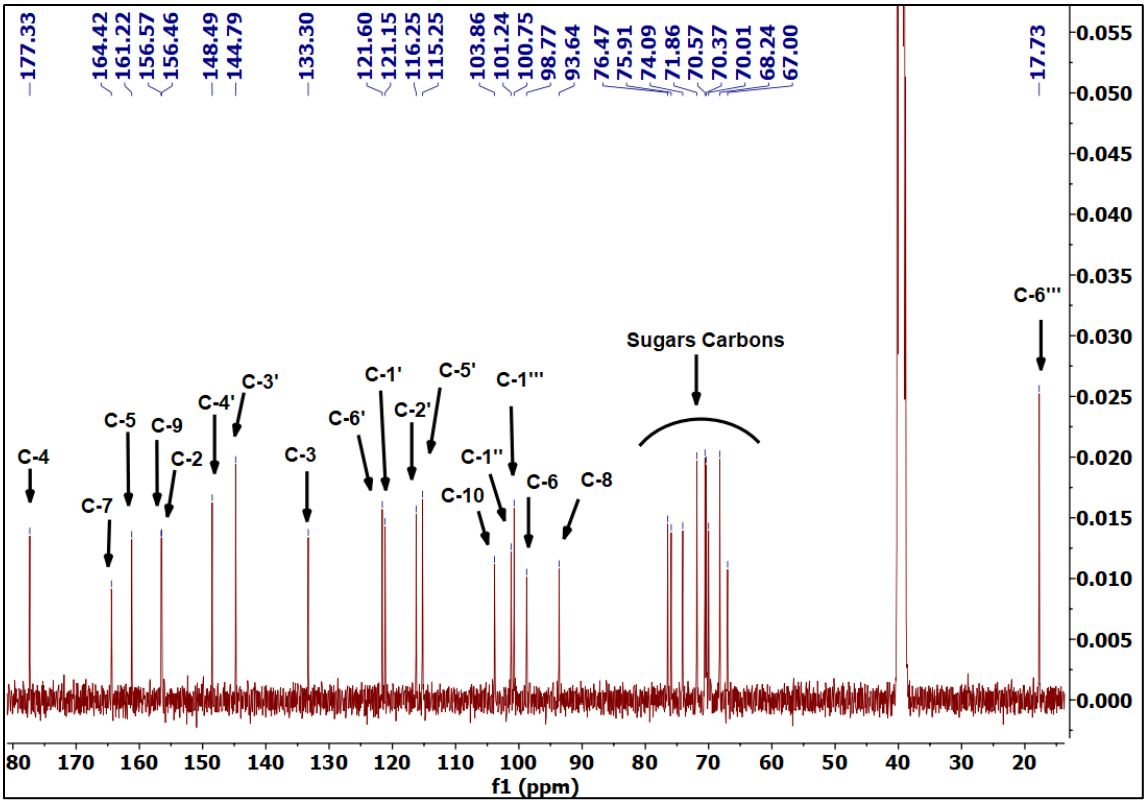

Fig. S15 ^{13}C NMR spectrum of compound **8** (DMSO-d_6, 125 MHz).

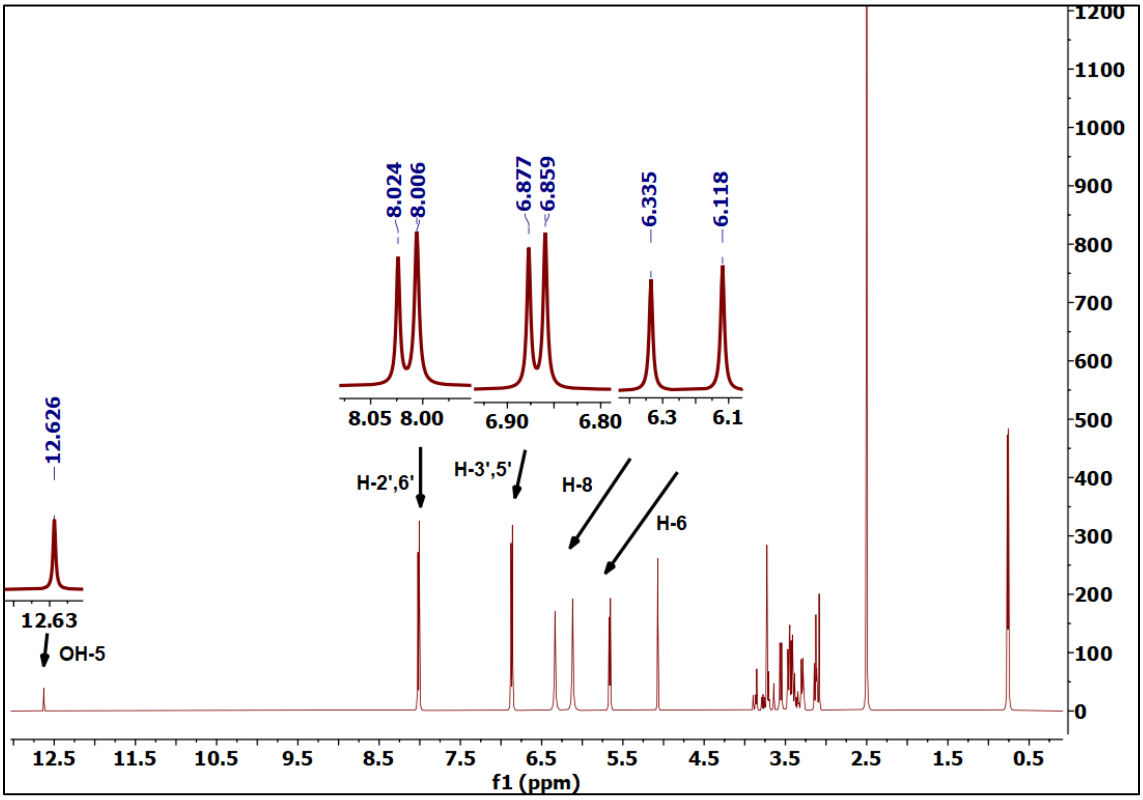

Fig. S17 ^{1}H NMR spectrum of compound **9** (DMSO-d_6, 500 MHz).

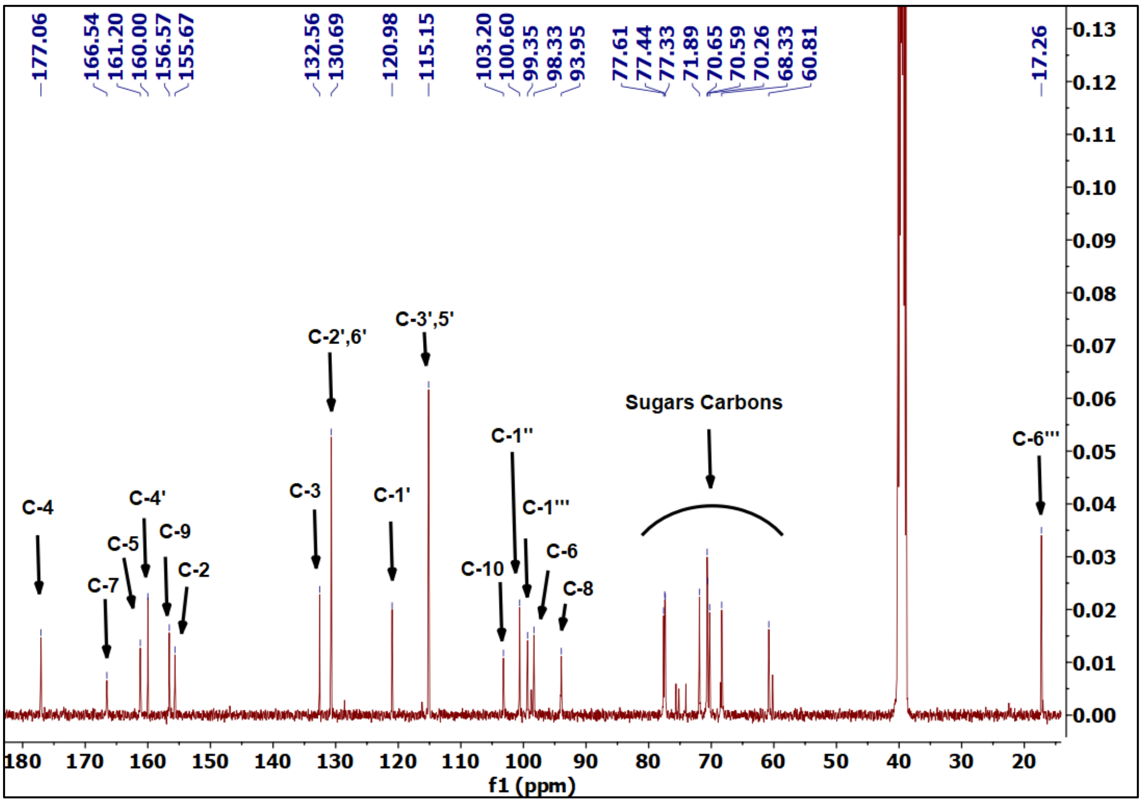

Fig. S18 ^{13}C NMR spectrum of compound **9** (DMSO-d_6, 125 MHz).

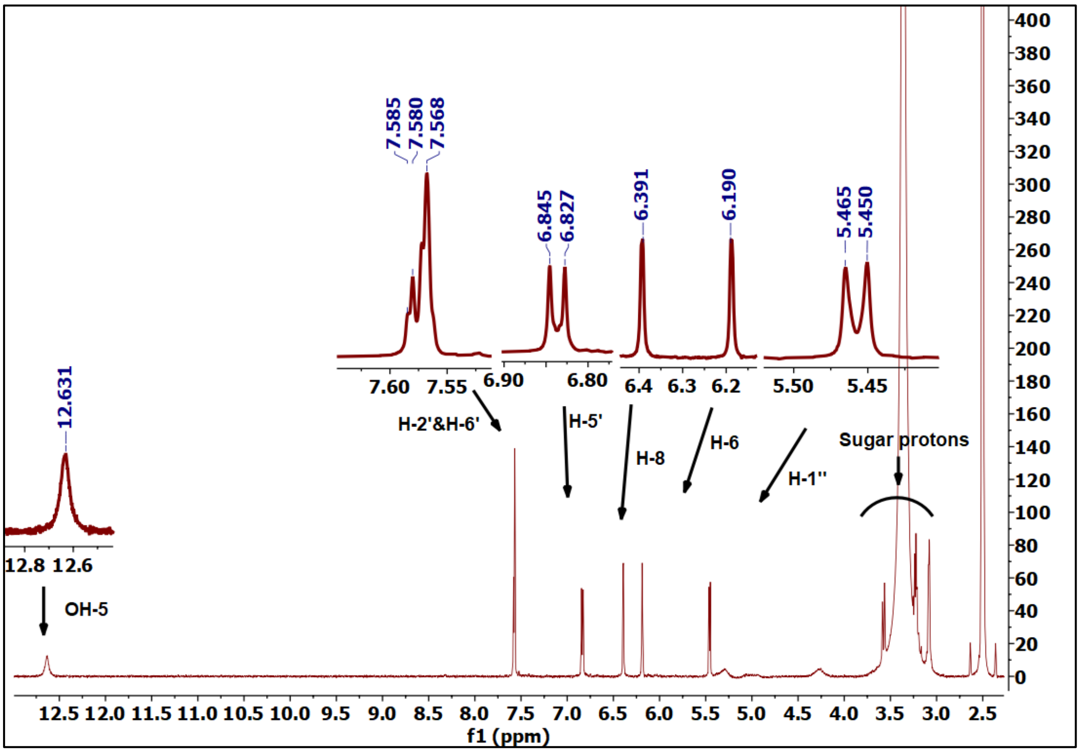

Fig. S19 ^{1}H NMR spectrum of compound **10** (DMSO-d_6, 500 MHz)

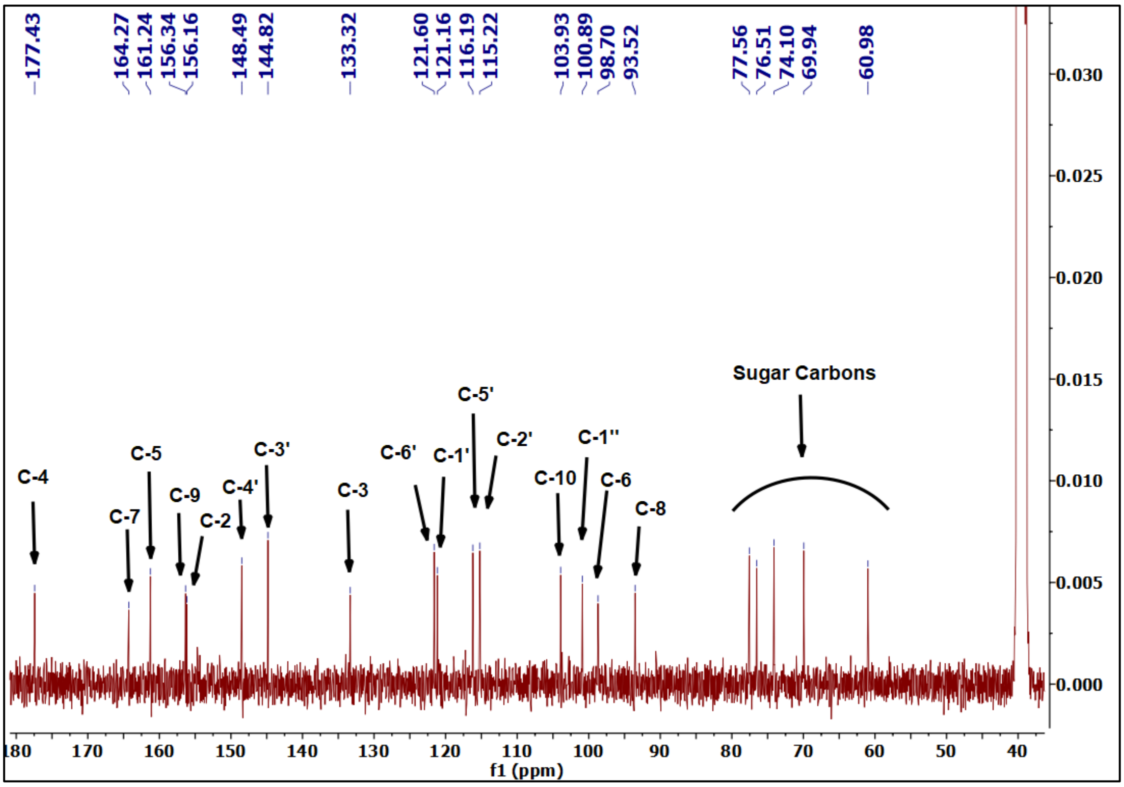

Fig. S20 ^{13}C NMR spectrum of compound **10** (DMSO-d_6, 125 MHz).

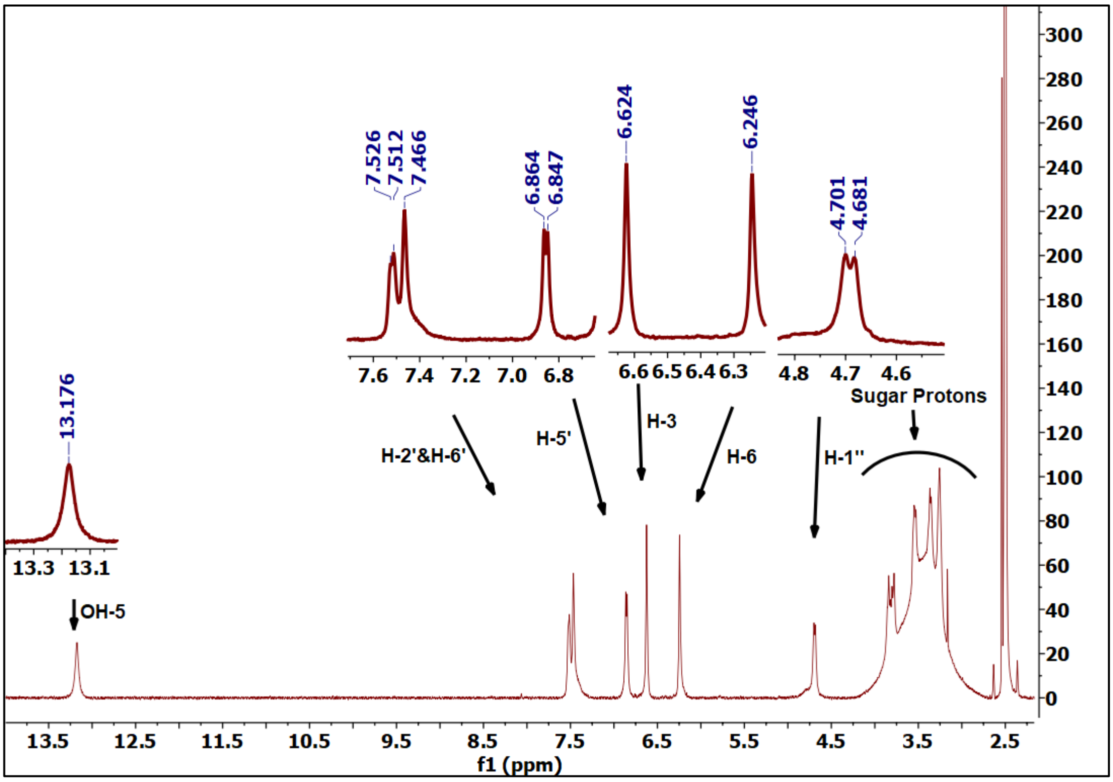

Fig. S21 ^{1}H NMR spectrum of compound **11** (DMSO-d_6, 500 MHz).

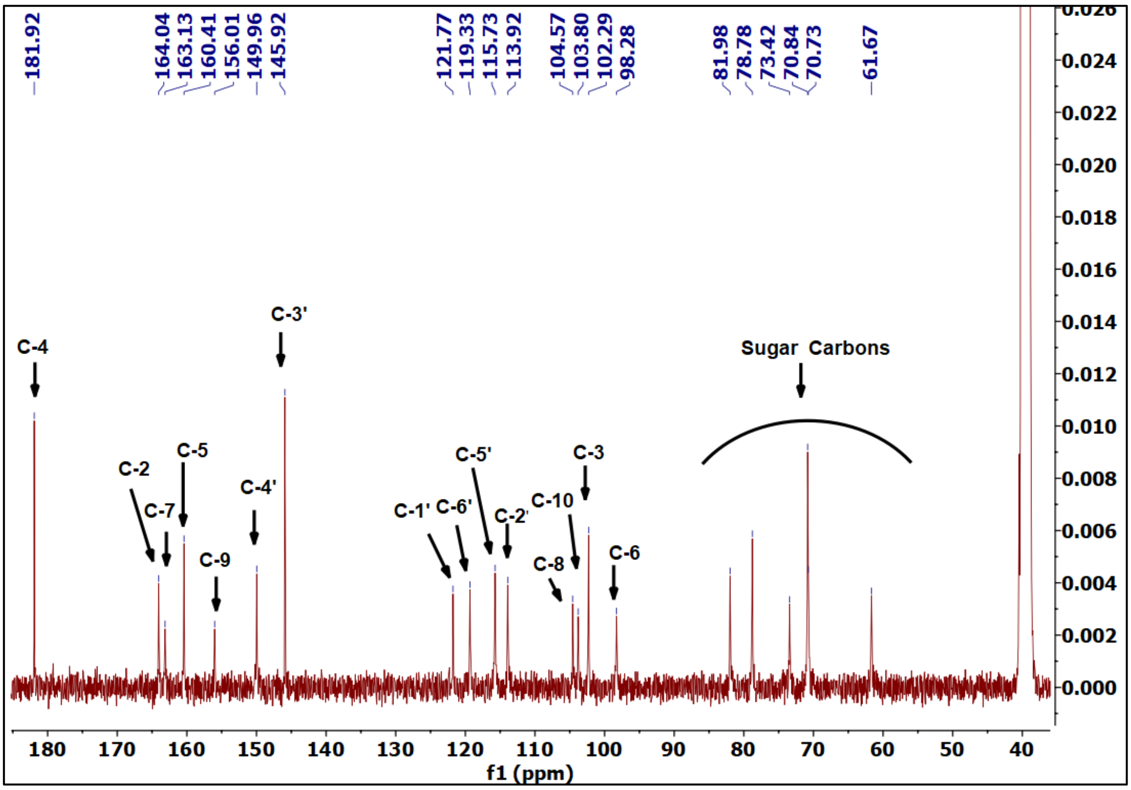

Fig. S22 ^{13}C NMR spectrum of compound **11** (DMSO-d_6, 125 MHz).

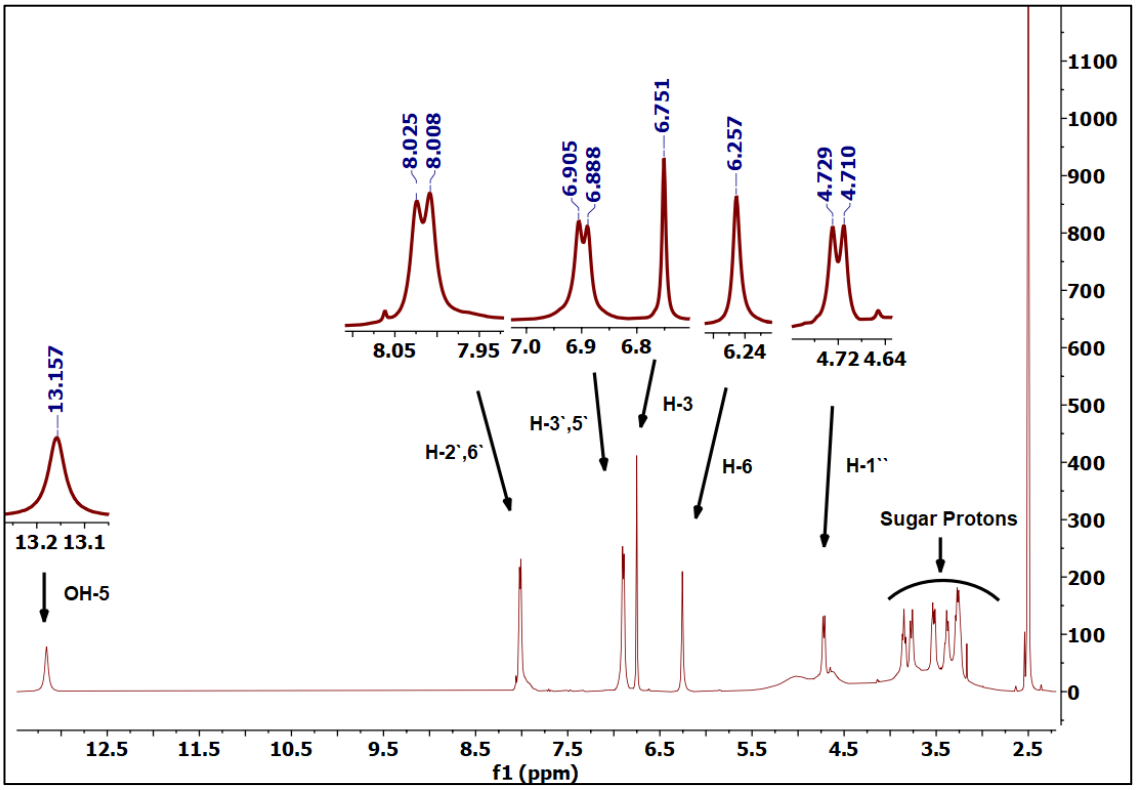

Fig. S23 ^{1}H NMR spectrum of compound **12** (DMSO-d_6, 500 MHz).

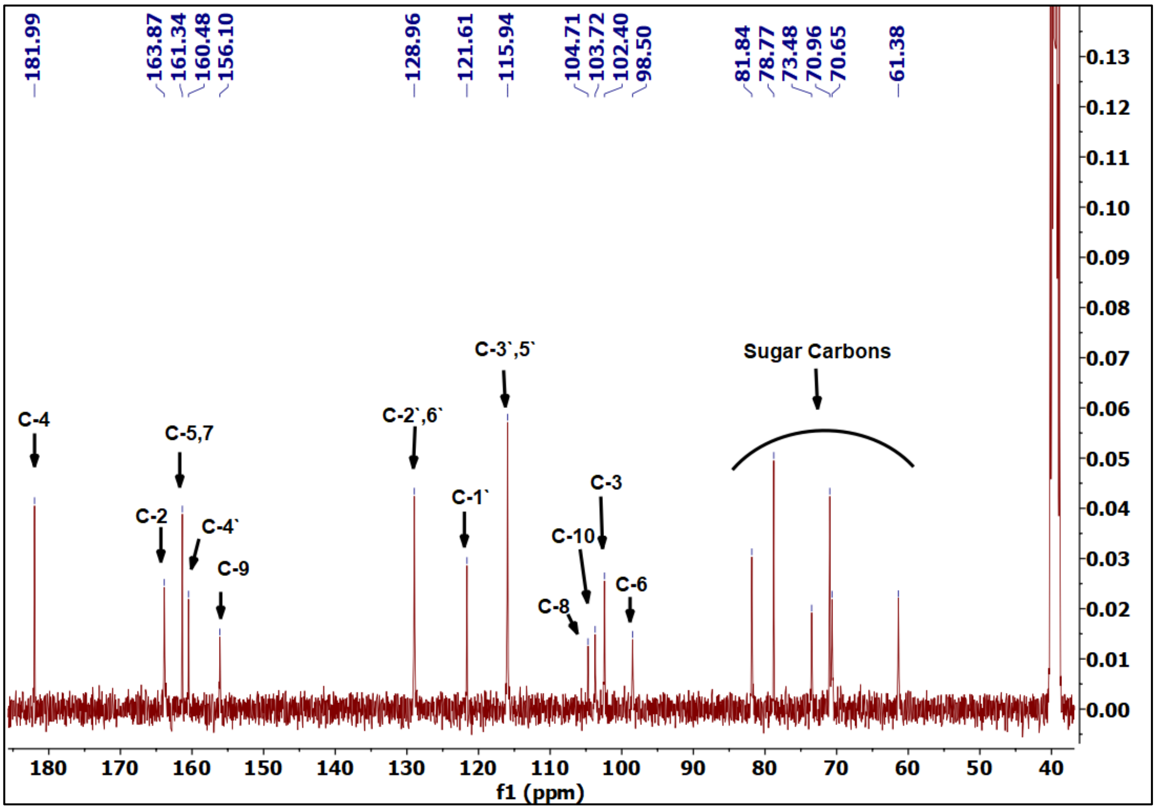

Fig. S24 ^{13}C NMR spectrum of compound **12** (DMSO-d_6, 125 MHz).

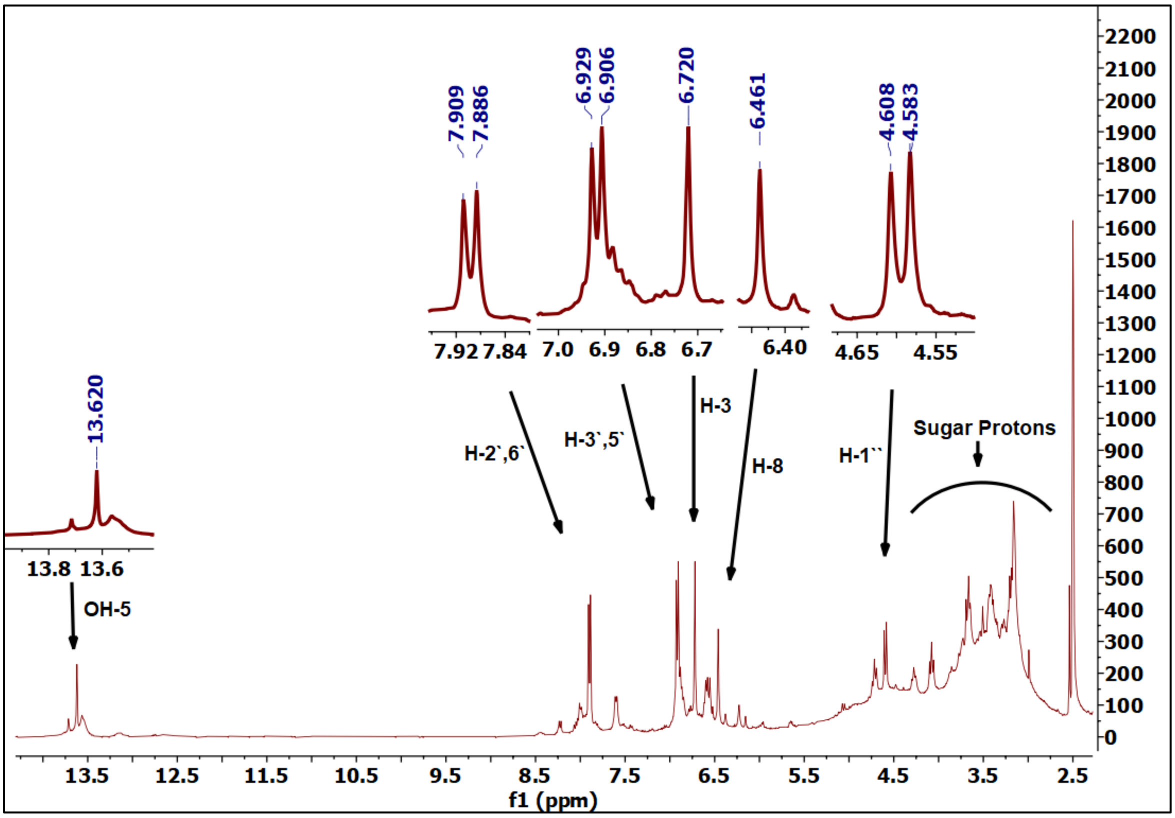

Fig. 25 ^{1}H NMR spectrum of compound **13** (DMSO-d_6, 400 MHz).

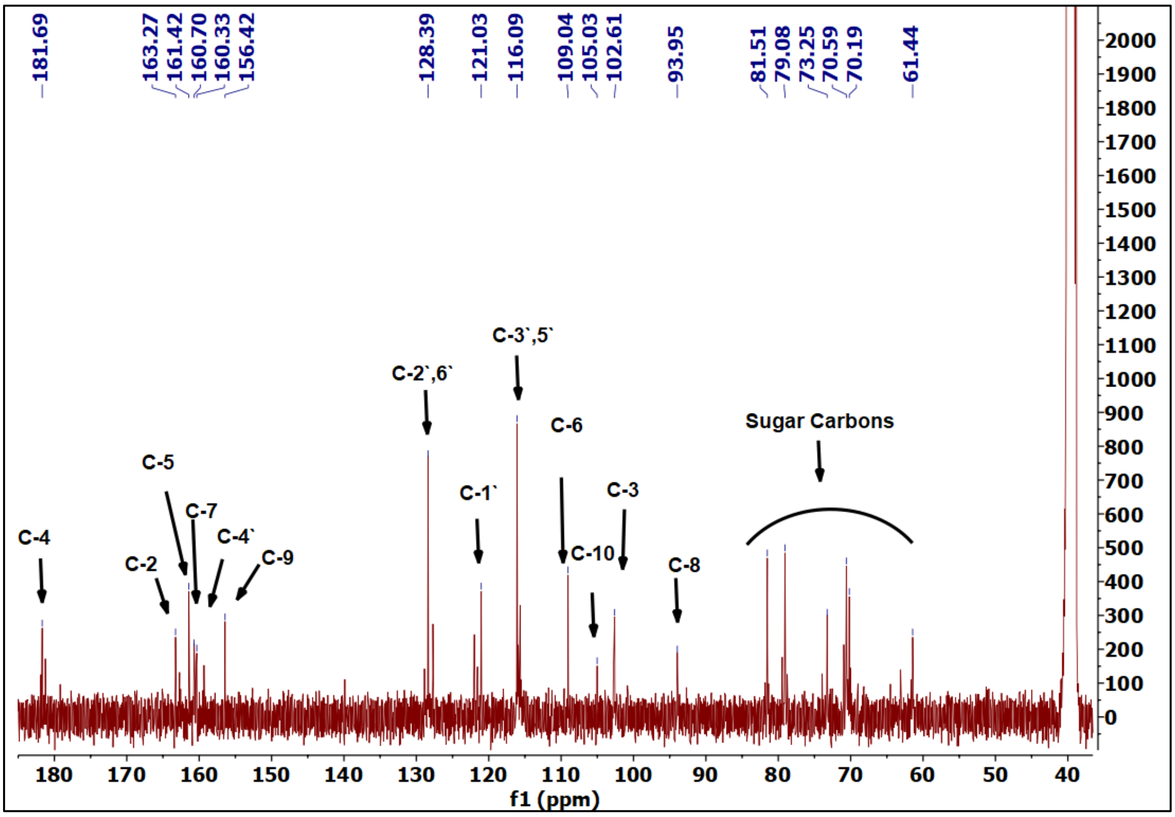

Fig. 26 ^{13}C NMR spectrum of compound **13** (DMSO-d_6, 100 MHz).

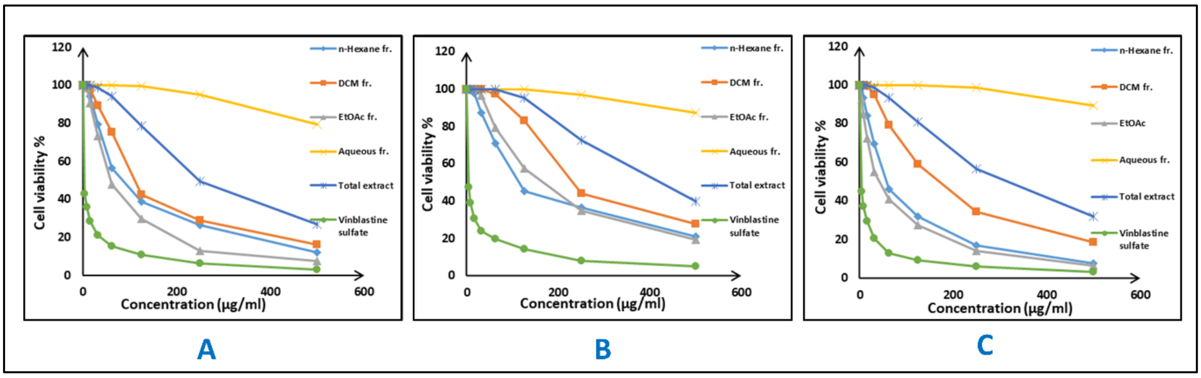

Fig. S27 Dose response (cytotoxicity) curve of the total extract and different fractions against HCT-116 (A), MCF-7 (B) and HepG-2 (C) cell lines.

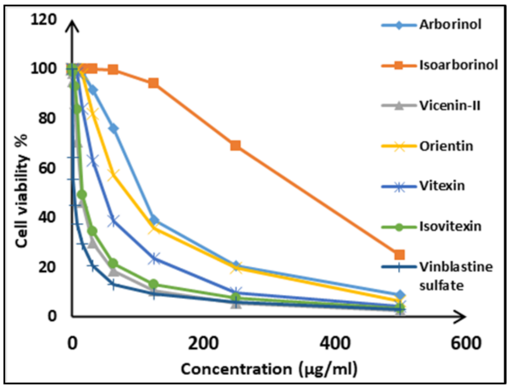

Fig. S28 Dose response (cytotoxicity) curve of some isolated compounds from *Dypsis pembana* leaves against HepG-2 cells.

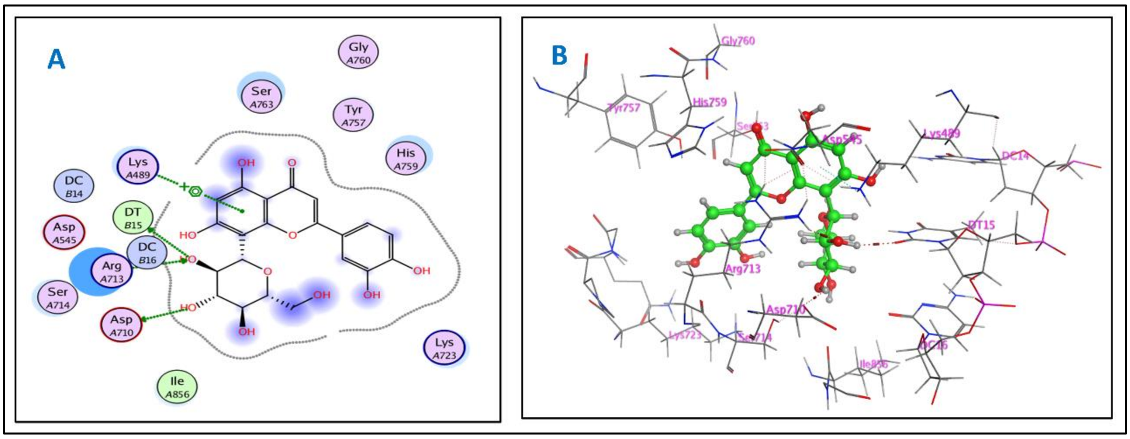

Fig. S29 2D (A) and 3D (B) interactions of compound **11** with topoisomerase IIα (PDB ID: 5gwk).

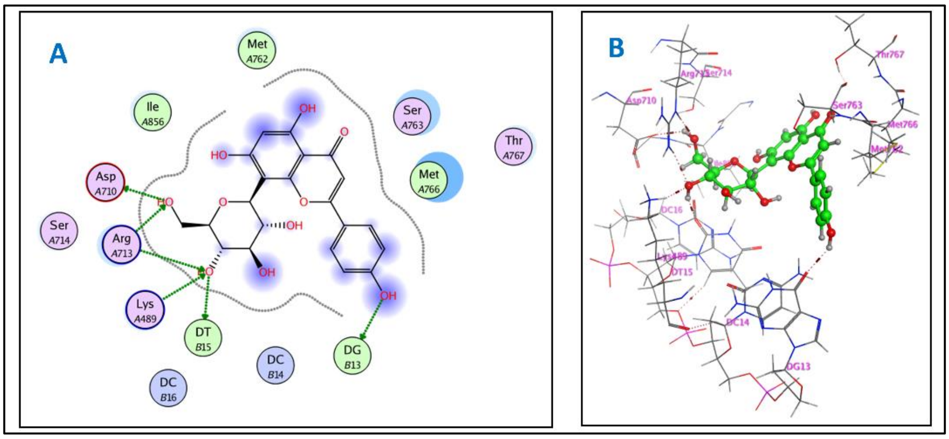

Fig. S30 2D (A) and 3D (B) interactions of compound **12** with topoisomerase IIα (PDB ID: 5gwk).

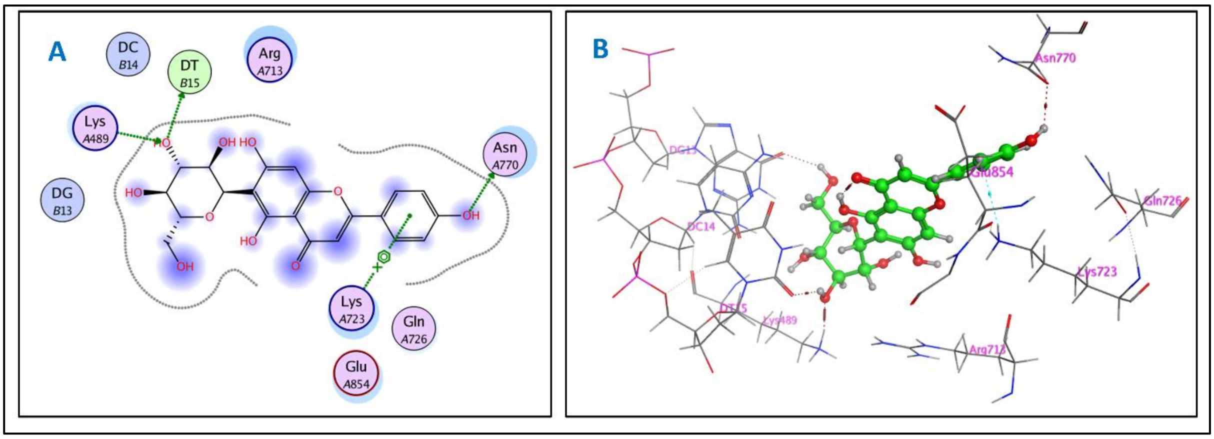

Fig. S31 2D (A) and 3D (B) interactions of compound 13 with topoisomerase IIα (PDB ID: 5gwk).

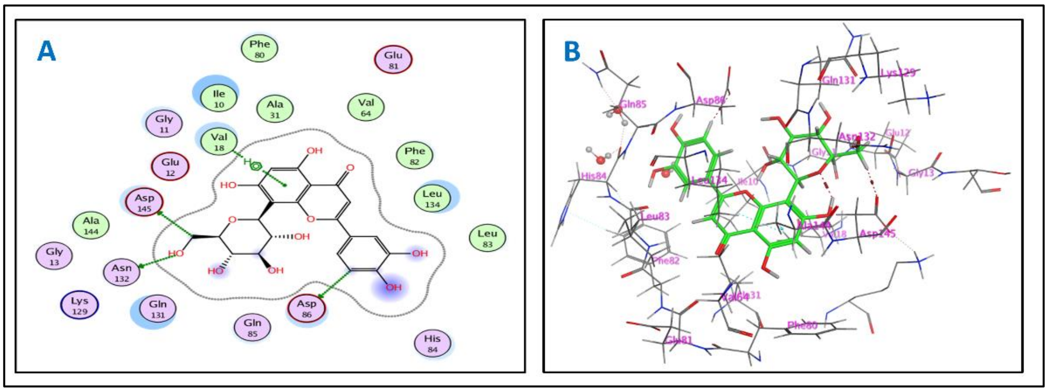

Fig. S32 2D (A) and 3D (B) interactions of compound **11** with cyclin-dependent kinase 2 (PDB ID: 3ti1).

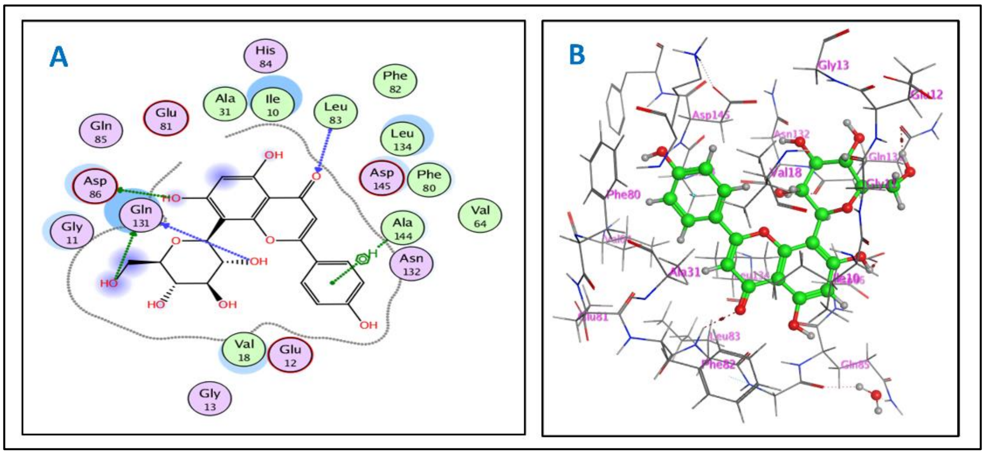

Fig. S33 2D (A) and 3D (B) interactions of compound **12** with cyclin-dependent kinase 2 (PDB ID: 3ti1).

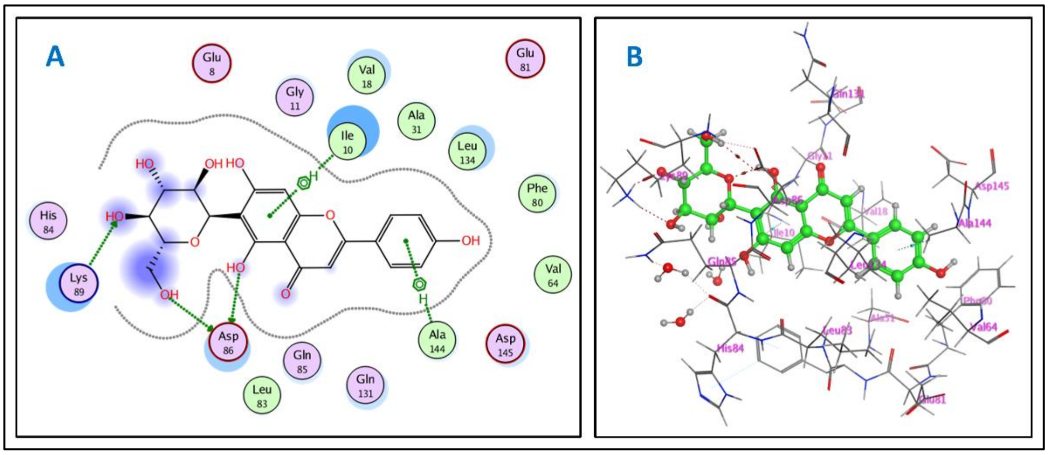

Fig S34 2D (A) and 3D (B) interactions of compound **13** with cyclin-dependent kinase 2 (PDB ID: 3ti1).

Table S2 Binding scores of the docking on topoisomerase IIα (PDB ID: 5gwk) and cyclin-dependent kinase 2 (PDB ID: 3ti1)

Sample	Topoisomerase IIα		Cyclin-dependent kinase 2	
	ΔG (Kcal/mol)	Amino acids involved in interaction	ΔG (Kcal/mol)	Amino acids involved in interaction
**Etoposide**	−7.67	Lys A723, Arg A713, Gly A462, Met A766, DG B13	-	-
**Sunitinib**	-	-	−7.62	Asp86, Glu81, Ile10, Ala144
**1**	−6.78	Met A766, Lys A723, DA B12, DG B13	−6.13	Ile10, Lys89
**2**	−7.24	Met A766, Lys A489, DG B13, DC B14	−6.19	Ile10, Lys89
**3a**	−6.45	Met A766, Lys A489, Arg A487, Glu A461, Arg A713, DG B13	−6.15	Asp86, Ile10, Glu8, Phe80, Leu83
**3b**	−7.38	Met A766, Gln A726, Arg A713, Lys A723, DG B13	−6.75	Ile10, Lys89
**4**	−7.29	Met A766, Lys A489, Lys A723, Arg A713, Asn A770, Ile A856, DG B13, DT B15	−7.48	Asp86, Ile10, Leu83, Glu8, Asp145, Gln131, Val18, Ala144
**5**	−6.84	Met A766, Lys A723, Arg A713, Asn A770, DG B13, DC B16, DT B15	−7.53	Asp86, Ile10, Leu83, Glu8, Asp145, Gln131, Val18, Ala144
**6**	−7.72	Met A766, Gly A462, Lys A489, Glu A461, DG B13, DT B15	−7.40	Asp86, Ile10, Leu83, Glu8, Val163
**7**	−8.52	Met A766, Gly A725, Lys A723, Arg A713, His A759, Glu A461, DG B13, DT B15, DC B14	−8.67	Asp86, Ile10, Leu83, Glu8, Asp145, Gln131, Glu12, Lys89, Ala144
**8**	−7.95	Met A766, Lys A489, Lys A723, Arg A713, Ser A714, Ile A856, DG B13, DT B15	−8.23	Asp86, Ile10, Leu83, Glu8, Leu298, Glu12, Gln131, Lys89, His84
**9**	−8.36	Met A766, Lys A489, Lys A723, Arg A713, His A759, Gly A725, DG B13, DT B15	−8.02	Asp86, Ile10, Leu83, Glu8, Asp145, Glu12, Gly13, His84
**10**	−7.68	Met A766, Lys A489, Lys A723, Arg A713, His A759, DG B13, DT B15, DC B16	−8.50	Asp86, Ile10, Leu83, Glu8, Asp145, Gln131, Glu81, Val18
**11**	−7.83	Met A766, Lys A489, Lys A723, Arg A713, Asp A710, DG B13, DT B15	−7.65	Asp86, Ile10, Leu83, Glu8, Asp145, Asn132, Val18, Glu12
**12**	−7.88	Met A766, Lys A489, Lys A723, Arg A713, Asp A710, DG B13, DT B15, DC B16	−8.31	Asp86, Ile10, Leu83, Glu8, Asp145, Gln131, Val18, Ala144
**13**	−7.51	Met A766, Lys A489, Lys A723, Arg A713, Asn A770, DG B13, DT B15	−8.12	Asp86, Ile10, Leu83, Glu8, Asp145, Gln131, Glu12, Lys89, Ala144
